# Microparticles from dental calculus disclose paleoenvironmental and palaeoecological records

**DOI:** 10.1002/ece3.11053

**Published:** 2024-02-23

**Authors:** Alessia D'Agostino, Gabriele Di Marco, Mario Federico Rolfo, Luca Alessandri, Silvia Marvelli, Roberto Braglia, Roberta Congestri, Federica Berrilli, Maria Felicita Fuciarelli, Angelica Ferracci, Antonella Canini, Angelo Gismondi

**Affiliations:** ^1^ Laboratory of Botany, Department of Biology University of Rome Tor Vergata Rome Italy; ^2^ Department of History, Culture and Society University of Rome Tor Vergata Rome Italy; ^3^ Groningen Institute of Archaeology University of Groningen Groningen The Netherlands; ^4^ Laboratory of Palynology and Archaeobotany‐C.A.A. Giorgio Nicoli Bologna Italy; ^5^ Laboratory of Biology of the Algae, Department of Biology University of Rome Tor Vergata Rome Italy; ^6^ Department of Clinical Sciences and Translational Medicine University of Rome Tor Vergata Rome Italy; ^7^ Laboratory of Human Ecology, Department of Biology University of Rome Tor Vergata Rome Italy; ^8^ Present address: PhD Program in Evolutionary Biology and Ecology, Department of Biology University of Rome Tor Vergata Rome Italy

**Keywords:** ancient landscape, paleoenvironment, plant ecology, prehistoric times, tartar, water sources

## Abstract

Plants have always represented a key element in landscape delineation. Indeed, plant diversity, whose distribution is influenced by geographic/climatic variability, has affected both environmental and human ecology. The present contribution represents a multi‐proxy study focused on the detection of starch, pollen and non‐pollen palynomorphs in ancient dental calculus collected from pre‐historical individuals buried at La Sassa and Pila archaeological sites (Central Italy). The collected record suggested the potential use of plant taxa by the people living in Central Italy during the Copper‐Middle Bronze Age and expanded the body of evidence reported by previous palynological and palaeoecological studies. The application of a microscopic approach provided information about domesticated crops and/or gathered wild plants and inferred considerations on ancient environments, water sources, and past health and diseases. Moreover, the research supplied data to define the natural resources (e.g., C_4_‐plant intake) and the social use of the space during that period. Another important aspect was the finding of plant clues referable to woody habitats, characterised by broad‐leaved deciduous taxa and generally indicative of a warm‐temperate climate and grassy vegetation. Other unusual records (e.g., diatoms, brachysclereids) participated in defining the prehistoric ecological framework. Thus, this work provides an overview on the potential of the human dental calculus analysis to delineate some features of the ancient plant ecology and biodiversity.

## Graphical abstract

Dental calculus analysis reveals evidence about past dietary ecology and plant diversity. This multi‐proxy study focuses on pollen and non‐pollen palynomorphs. We detected plants referable to woody habitats and warm‐temperate climates. Clues about past ecological and environmental issues of Central Italy were found.

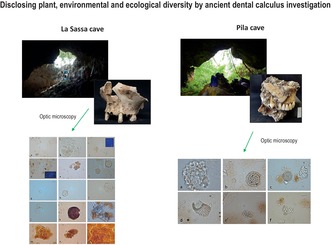

## 1 INTRODUCTION

Evidence from ancient dental calculus may provide fascinating new insights into environments, plant biodiversity and ecological contexts existing during prehistoric times (Carra et al., 2022; Cristiani et al., 2016, 2021; Goude et al., 2019; Hardy et al., 2016, 2017, 2018; Henry et al., 2011; King et al., 2017). The organic particles included in this archaeo‐anthropological matrix have various origins; thus, various studies have been carried out to investigate its composition. The first research approach on tartar was focused on extraction and identification of microfossils, such as starch and phytoliths, because they could be used to trace back to the presence and use of specific plants. More recently, the analysis of proteins, plant secondary metabolites and DNA from calculus has been also explored (Goude et al., 2019; Hardy et al., 2012; Henry et al., 2011; King et al., 2017; Norström et al., 2019; Ottoni et al., 2021; Wang et al., 2022; Warinner et al., 2014). In this context, as for all ancient remains, one of the major debated issues is related to the efficiency of decontamination. This is a key step to obtain authentic results, whatever the target to be investigated (i.e., microfossil, ancient DNA, chemical compound). Over the years, researchers have developed several cleaning procedures, as well as novel approaches (e.g., Raman spectroscopy, X‐ray photoelectron spectroscopy, Next Generation Sequencing) capable of analysing both the organic and inorganic constituents of the trapped materials (i.e., minerals, oral microbiota, starch) (Ottoni et al., 2021; Radini et al., 2019; Soto et al., 2019). Despite that, the reconstruction of non‐dietary aspects from calculus records is still challenging. As reported by Radini et al. (2017), oral breathing continuously occurs during the lifetime of an individual; together with the air, the particles (up to 70 μm) can be easily aspirated and, later, embedded in dental calculus. Plant and animal debris can be transported up to the mouth both naturally by wind (e.g., pollen, hairs) and/or human activities, such as flour grinding, woodworking, eating, drinking and bringing dirty hands to the mouth (e.g., starch, diatoms, mineral grit, fibres, micro‐charcoal fragments), impressing a partial environmental fingerprint into tartar.

Geographic and climatic variability influences the distribution of phyto‐associations and, in the past, it has affected the natural landscape and, consequently, the subsistence strategies of prehistoric communities living there. Thus, the aforementioned particles, potentially traceable in tartar, may be considered environmental multiproxies reflecting or testifying elements present in the settlement area and cultural habits evolved in relation to plant natural resources.

Natural caves have represented typical places for burial during Prehistory, although they were rarely characterised by an easy entrance. Among them, La Sassa (Sonnino, Latina) and Pila (Pozzaglia Sabina, Rieti) constitute precious depositories of prehistoric cultural heritage in Central Italy.

**FIGURE 1:**
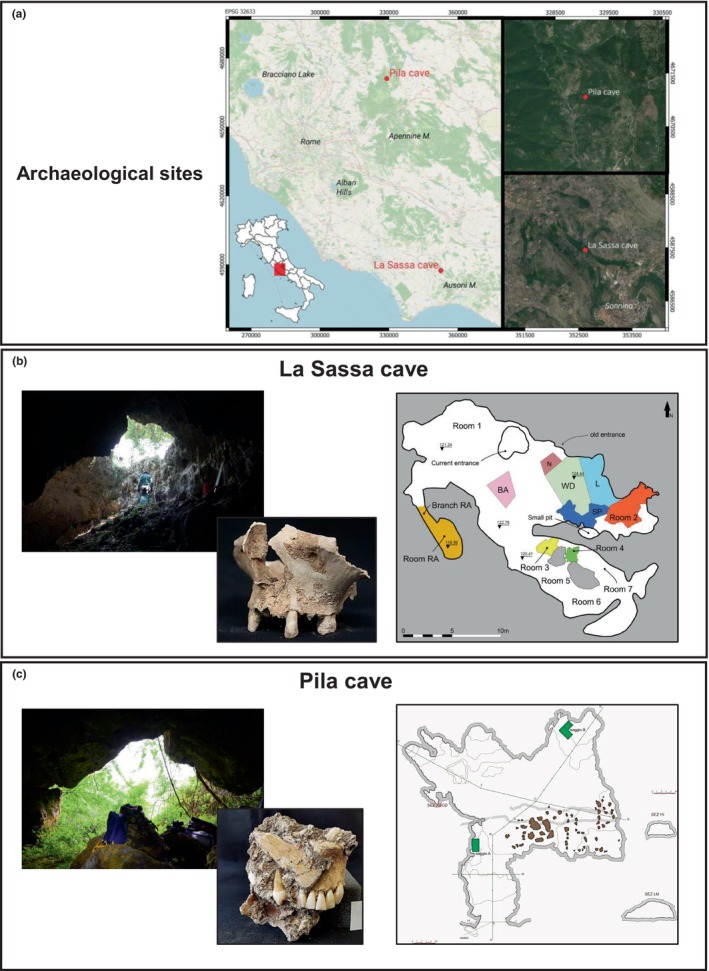
Archaeological sites. Geographical localisation of the studied archaeological sites (Latium, Central Italy) (red dots; panel a). For La Sassa cave (coordinates of the entrance WGS84, 41°25′30″ N, 13°14′11″ E; Sonnino, LT; panel b) and Pila cave (coordinates of the entrance 42°10′26″ N; 12°55′46″ E; Pozzaglia Sabina, RT; panel c), pictures of the current entrance (taken from the internal rooms), a representative human skeletal remain, and the relative planimetry were shown. Photos by A. Ferracci and A. D'Agostino.

Since 2016, four archaeological campaigns have been carried out at La Sassa cave in the Ausoni Mountains. The cave was used both as a human burial place, from Copper Age (CA) to Middle Bronze Age (MBA), and for performing possible ritual activities, in the Middle Bronze Age (Figure 1). The chronology is based both on ceramic chronology (relative) and on radiocarbon dates (absolute). Previous evaluations allowed to determine a minimum number of individuals buried at the site 20, a distance average walking time from the nearest settlements of about 50 min, and a local mobility relative to an area of a few kilometres around the cave (Alessandri et al., 2021; Romboni et al., 2023).

In September 2019, the first systematic survey and archaeological excavation campaign of Pila cave took place, in order to verify the presence of human burials ascribable chronologically to CA and the Early Bronze Age (EBA). Pila cave is located on the western side of the Eastern area of the Sabini mountains. The cave developed for 74 m and, like other cavities present in Sabina, it likely was a place of worship and/or burial (Figure 1) (Piro et al., 2018). So far, no prehistoric settlements have been reported in this area. The human skeletal series found in the test pit (Saggio B) carried out in the innermost chamber of the cave consisted of remains, especially teeth, arranged in an unorderly manner without an evident anatomical connection (perhaps the result of skeletal reduction practices or secondary manipulations). Typical elements of both male and female funerary equipment were also found in the cave (i.e., potsherds, flint arrowheads, a perforated calcite bead and a cylindrical loom weight).

The stratigraphy of both caves refers to a period in which climatic/environmental changes have influenced the landscape evolution in Central Italy. Palynological analyses have defined a vegetational profile characterised by mixed‐oak forests, associated with *C. betulus*, *O. carpinifolia*/*C. orientalis*, *Fagus* and *Alnus* sp. (Bellotti et al., 2016; D'Agostino, Di Marco, Marvelli, Marchesini, Martínez‐Labarga, et al., 2022; Di Rita et al., 2010, 2018; Magri & Sadori, 1999; Mercuri et al., 2002; Peyron et al., 2011). In addition, a significant decrease in forest covering, caused by climatic drying, has been recorded (Doorenbosch & Field, 2019; Mercuri & Sadori, 2012). At the same time, topographical conformation and presence of water basins in these areas might have favoured the development of various plant associations, as documented by the intensification of species typical of damp environment and Monilophyta. To date, no information about plant diversity and human ecology for La Sassa and Pila prehistorical contexts is known, the question about the existence of wild and/or domestic plants and their putative use by the ancient communities of Central Italy remains open. Thus, to fill this gap in the knowledge, we decided to explore whether the micro botanical particles in the dental calculus of the individuals buried in these archaeological sites could be used for reconstructing these aspects. Indeed, no macro botanical records have been found to the sites and palynological reconstructions based on cave deposits are complex and arduous, due to the great number of stochastic local depositional and post‐depositional events influencing pollen taphonomy (D'Agostino, Di Marco, Marvelli, Marchesini, Martínez‐Labarga, et al., 2022). So here, we collected microscopic ancient evidence contributing to provide further insights about the presence of plant taxa in the studied environments, to grow the general body of evidence already reported by previous palynological studies and to clarify their potential role for/relationship with the people living in the Italian Central Apennines.

## 2 MATERIALS AND METHODS

Human skeletal remains, radiocarbon calibrated at Copper‐Middle Bronze Age (see Appendix S1) and preserved at the Laboratory of the Department of History, Culture and Society at the University of Rome Tor Vergata (Italy), were analysed to check presence of teeth and tartar. Before sampling, we observed and documented supragingival dental calculus deposits (Figure 1).

A total of 117 mineralised plaque samples were undergone to optical microscopy analysis for detecting microparticles. Seventy‐eight of them (LSC samples) were collected from human skeletal remains unearthed from La Sassa cave and identified as belonging to single individuals (15 samples; mentioned as A–Q) or isolated teeth (63 samples; named 1–63). The remaining 39 (PC samples), consisting of tartar from 9 single individuals (A–I) and 30 isolated teeth were obtained starting from remains dug up during archaeological excavations at Pila cave. In detail, sampled teeth were codified according to the International Dental Federation tooth notation ISO 3950 (2009).

Each step of the analysis was carried out in the cleanroom facilities of the Department of Biology of the University of Rome Tor Vergata, following our published lab standard protocols (D'Agostino et al., 2020, 2021). There, the areas are used exclusively for dental calculus investigation and an intensive cleaning regime was applied before and during the processing. Laboratory contamination checks are regularly performed on all workspaces and supplies and all the steps are conducted under a sterile vertical laminar flow hood, to prevent any potential contamination and to exclude post‐depositional intrusion. The absence of plant and other micro debris in laboratory reagents, water and materials is always monitored.

The soil still adhering to the external part of the mineralised plaque was gently removed using a fine sterile needle, under a stereomicroscope (Leica ZOOM 2000, Leica, Bufalo, NY, USA). Then, light calculus deposits were removed from tooth enamel, by an autoclaved dental pick on an aluminium foil and placed in sterilised micro‐centrifuge tubes. Taking into consideration the work of Farrer et al. (2021) and our previous evidence (D'Agostino, Di Marco, Marvelli, Marchesini, Rizzoli, et al., 2022), tartar was treated with ultraviolet radiation (UV) for 10 min and immersed in 5% sodium hypochlorite (NaClO), to minimise the exogenous content of the outer surface of the ancient calculus flakes. Afterward, samples were washed twice with ultrapure sterilised water and dried out at 37°C. Preceding the decalcification protocol, 45 randomly selected human calculi were washed by sterile water, which was examined at optical microscopy to confirm the efficacy of the sterilisation method. No microfossils were detected after decontamination.

For each sample, 0.5 mL of 0.2 M hydrochloric acid was added for 24 h, in agitation. The pellet resulting from this step was washed three times and mounted on a glass slide in a water‐glycerol solution (1:1, v/v) for identifying microparticles. The latter were observed and subjected to morphological analysis by an optical microscope (ZEISS Axio Observer 7, Zeiss, Jena, Germany) equipped with polarised filters and Zen imaging sofware 2.6, operating at different magnifications. The recovered microremains (i.e., starch granules, palynomorphs, non‐pollen palynomorphs) were classified based on morphology and described using conventional nomenclature (e.g., Adojoh et al., 2019; Ahituv & Henry, 2022; ICSN, 2011; Neumann et al., 2019), specific literature papers, our reference collections and public databases of wild and domestic plant species (e.g., PalDat, 2019).

## 3 RESULTS

Overall, the present research disclosed the presence of two starch morphotypes and several pollen and non‐pollen palynomorphs (henceforth NPPs) in the studied calculus samples. NPPs included, for example, remains of fern spores, parasite eggs, palynodebris elements (e.g., structured and structureless organic matter, cuticles, brachysclereids, phytoliths and wood fragments), microalgae and/or Radiolaria fragments and testate amoebae (Appendix S2), which will be discussed below.

### 3.1 La Sassa cave

#### 3.1.1 Starch

Upward of 1366 starch granules were retrieved in a good state of preservation from 31 calculus samples out of a total of 78 (see Appendix S2‐Table S1). They were grouped into two morphological types, according to shape, size, presence of *lamellae* and *hilum* and aggregation level and described using the International Code for Starch Nomenclature (ICSN, 2011). The diagnostic features for each morphotype are reported in Table 1.

**TABLE 1:** Taxonomic identification and morphological description of the observed microremains.

Taxonomic identification	Description of the observed morphological elements
Starch morphotype	
Triticeae Dumort. tribe	The large starches appeared oval to sub‐round in 2D shape (28–45 μm in length; 17–36 μm in width) with visible *lamellae* and a central *hilum*, while the small ones (≤11 μm in diameter) were spherical.
Panicoideae Link. subfamily	Single oval to polygonal granules. They showed a centric *hilum*, perpendicular extinction cross, and evident central fissures. This morphotype was 12–29 μm in length and 10–17 μm in width.
Pollen type and spores	
Asteroideae	Palynomorphs spheroidal in equatorial view (equatorial axis of 23 μm) with peculiar echinate ornamentation of the exine.
Brassicaceae or Oleaceae	Spheroidal morphology (1–30 μm in polar view; colporate) with a reticulate ornamentation of the exine.
*Ostrya carpinifolia* Scop. or *Betula* L.	Triporate, isopolar, and psilate pollen, 23 μm long in equatorial view.
*Alnus* spp.	The microdebris was oblate in shape with typically vestibulate aspidate (protruding) pores along the equatorial plane (stephanoporate) (22 μm long).
Cupressaceae	The morphology appeared spherical (with polar and equatorial axes of 24 μm) with a star‐like protoplast and inaperturate.
Fagaceae (i.e., *Quercus* deciduous)	Pollen grains were single, prolate, isopolar, tricolpate, with long and narrow colpi, and elliptic in equatorial view (polar axis 17–25 μm long).
*Tilia*	Monad, tricolporate in polar view, planaperturate (that is the apertures are situated in the middle of the sides in polar view) grains, with lens‐shaped bodies located beneath the aperture (colporus) and presenting a medium size (24 μm in polar view).
Poaceae spontaneous group	The pollen was spherical (apolar) and monoporate (size: 77 μm in diameter; 30 μm in equatorial view).
*Aster*	Spheroidal (29 μm in equatorial view) and 3‐zonocolporate grain.
Cichorieae	Spheroidal shape, tricolporate aperture condition, lophate and echinate ornamentation, and 22 μm of dimension in polar view.
*Trifolium*	Subprolate pollen in equatorial view (24–26 μm) with scabrate ornamentation of the exine.
Fabaceae	One microremain was 3‐colporate, prolate, psilate exine, with 38 μm of length in equatorial view.
Ulmaceae (e.g., *Ulmus* sp.)	This palynomorph possessed spheroidal shape and verrucate exine (32 μm in polar view).
Pteridophytes‐monolete spore	The microremain exhibited psilate sculpture and was 35 μm long in equatorial view.
Polypodiaceae spore (*Polypodium* L.)	The morphology consisted in a bean‐shaped (59 μm in equatorial view), bilaterally symmetrical, and with a verrucate exine sculpture.
Pteridophyte megasporangium	The microdebris appeared brownish in colour and ovoid in shape (109.9 μm in in length and 104.2 μm in width), with 3 megaspores (49–44 μm in in length and 42–41 μm in width) clearly visible inside.

##### Morphotype I

**FIGURE 2:**
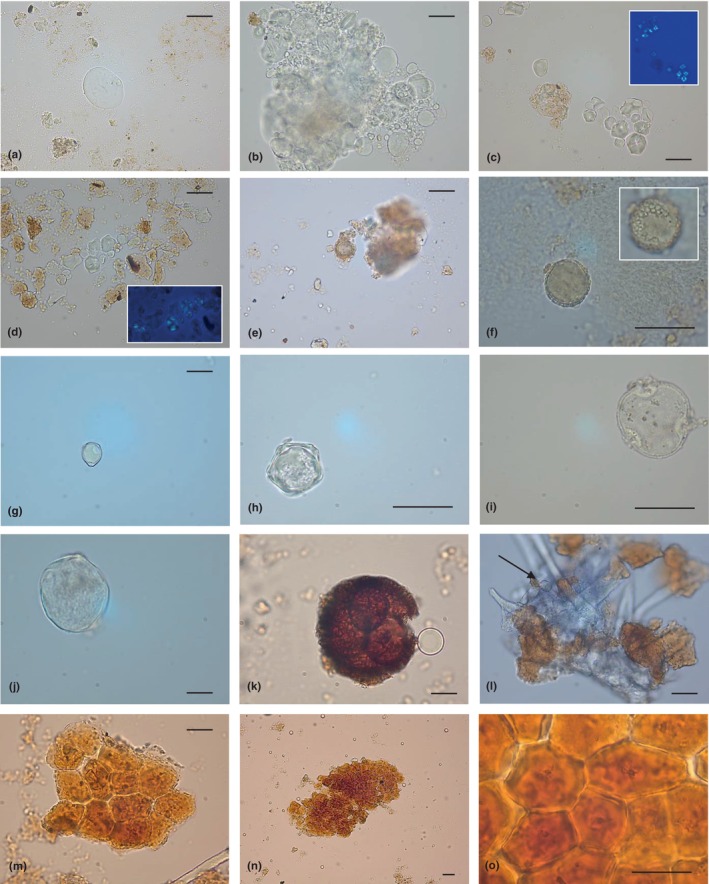
Mosaic of selected plant microparticles recovered in dental calculus samples from La Sassa cave. Some of the images captured by optic microscopy were shown. Triticeae starch (a); aggregate of Triticeae starch granules (b); Panicoideae starch granules, still adhering to calculus flecks, and relative polarised images (c and d); Asteroideae pollen (e); Brassicaceae/Oleaceae pollen grain emerging from undissolved dental calculus (f); Betulaceae pollen grain (g); *Alnus* spp. pollen (h); *Tilia* sp. pollen (i); Poaceae spontaneous group pollen (j); sporangium (k); fragment of plant epidermis with visible trichomes (the arrow indicates a polyhedral starch) (l); brachysclereid aggregate (m); brachysclereid aggregate at different magnifications (n and o). The scale bar indicates 25 μm (except for panel n: 55 μm).

More than 857 starch granules showed a morphology coherent with those of Triticeae Dumort. tribe (Appendix S2‐Table S1; Figure 2a,b). Usually, it is a common condition in caryopses of cereals, such as *Hordeum* sp. L. and *Triticum* sp. L. In some cases, grains appeared altered and/or in the form of lumps. Noteworthy, upward of 843 starches were counted only in the LSC F sample (Appendix S2), sometimes in the form of aggregates.

##### Morphotype II

Starch granules of this typology (>476), or groups of them, were embedded in different calculus samples (Appendix S2‐Table S1; Figure 2c,d). They occur in seeds of grasses belonging to the Panicoideae Link. subfamily. Several plant species are related to this taxonomic group (e.g., Paniceae tribe: *Setaria* sp. P. Beauv., *Panicum* sp. L.) and their starches overlap in size and shape, making a lower taxonomic identification challenging.

Thirty‐three undiagnostic starches, with no identifiable features, were found (indicated as not determined in Appendix S2). Together with the grinding process, cooking procedure in water and/or chewing, the oral bacterial activity might have changed their shape. Indeed, it is known that many oral streptococcal commensal species can adhere to starch granules and bind to salivary amylase. The latter would facilitate bacterial nutrition by releasing glucose from dietary starch for energy production (Butterworth et al., 2011; Nikitkova et al., 2013; Scannapicco et al., 1990).

#### 3.1.2 Pollen and spores

Pollen nomenclature used in this paragraph mostly follows Berglund and Ralska‐Jasiewiczowa (1986), Faegri and Iversen (1989) and Moore et al. (1991). The morphometric parameters were in accordance with the Palynological Database (PalDat, 2019) and described in detail in Table 1.

In total, 16 palynomorphs and one megasporangium with spores were found in the calculi from La Sassa cave (Appendix S2‐Table S1).

Two pollen grains were classified as not determined, as lacking distinctive diagnostic features. One sample showed 3 palynomorphs cautiously associated with Asteroideae type (Figure 2e), due to the absence of further peculiar characters.

Another ancient grain displayed a morphology tentatively ascribed to Brassicaceae or Oleaceae pollen types (Figure 2f) (Erdtman, 1986; Jin‐tan, 1982; Khalik et al., 2002).

Calculi from La Sassa cave also revealed the presence of two pollen attributed to the Betulaceae family (Figure 2g). One of them showed morphological features commonly occurring in *Ostrya carpinifolia* Scop. (Subfamily Coryloideae) and *Betula* L. (Subfamily Betuloideae) pollen (Halbritter, 2016; Mäkelä, 1996), while the appearance of the second one (Figure 2h) was reminiscent of palynomorphs from *Alnus* spp. (Faegri & Iversen, 1989), as they are peculiar in morphology within the Betulaceae.

Three microremains showed a morphology widespread in several species of Cupressaceae.

Other three pollen grains were closely similar to those produced by different species of Fagaceae (more specifically *Quercus* deciduous type), whose morphologies are not distinguishable as overlapping in size, shape and ornamentation (Denk & Tekleva, 2014; Grímsson et al., 2015).

In one calculus sample, a monad was identified with high probability as pollen of *Tilia* sp. L. (Malvaceae, Subfamily Tilioideae) (Figure 2i) (Halbritter et al., 2021).

The morphology of another ancient pollen typically resembled that of the Poaceae spontaneous group (Figure 2j) (Perveen, 2006); however, a more specific systematic recognition is difficult.

Finally, a Pteridophyte megasporangium (Figure 2k) was found in sample LSC O (Appendix S2‐Table S1). Unfortunately, a lower taxonomical identification would be risky.

#### 3.1.3 Non‐pollen palynomorphs (NPPs)

This section includes 22 palynodebris elements, or rather structured elements, such as plant cuticles and cells (Appendix S2‐Table S1).

A sample deriving from one isolated tooth showed a fragment of plant epidermis still preserving several unicellular conical trichomes. In Figure 2l, this tissue is shown embedded in an undissolved calculus matrix, also containing a polyhedral starch (black arrow).

In the other two individuals, 21 brachysclereid aggregates were observed (Figure 2m,n). Also called stone cells, brachysclereids are isodiametric sclerenchyma cells, formed by secondary deposition of lignin on the cell wall. In detail, our ancient micro debris corresponded in morphology and appearance to stone cell clusters typically occurring in the pith, cortex and bark of many stems and certain Rosaceae fruits and false fruits (such as *Pyrus* sp. L., *Prunus* sp. L., *Crataegus* sp. L. and so forth) (Barclay, 2007; Lin et al., 2022; Smith, 1935). The single sclereids looked greatly irregular and/or rectangular, yellowish in colour and with a narrow central cavity (clearly visible in the magnification reported in Figure 2o).

### 3.2 Pila cave

#### 3.2.1 Starch

**FIGURE 3:**
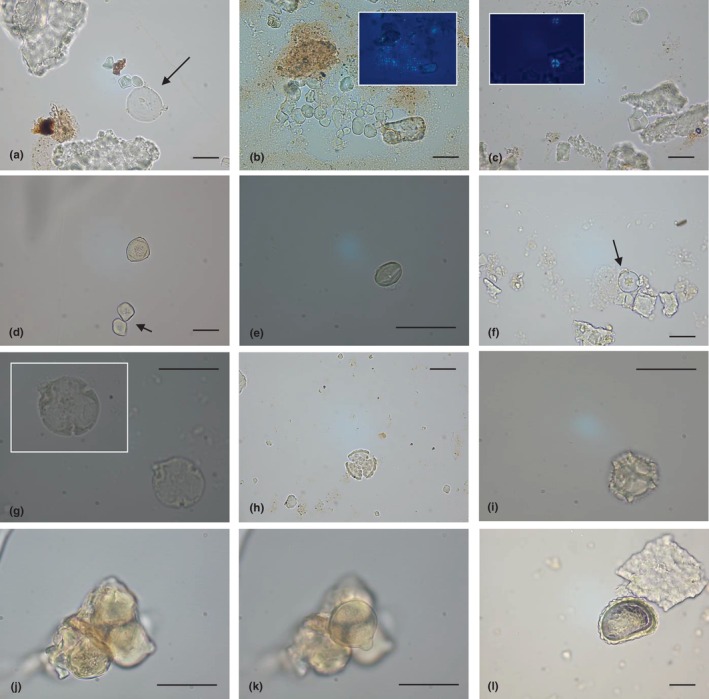
Plant microparticles retrieved from human calculi of Pila cave. Representative images obtained by optic microscopy analysis were shown. Triticeae starch (Morphotype I, indicated by the arrow) with three starch granules belonging to Morphotype II (a); aggregate of Panicoideae starch granules, still adhering to calculus flecks, and relative polarised image (b); Panicoideae starches and relative polarised image (c); Betulaceae pollen (the arrow indicates two polyhedral granules) (d); Fagaceae pollen (e); Cupressaceae pollen grain (indicated by the arrow), emerging from undissolved dental calculus (f); *Tilia* sp. pollen (g) Asteraceae pollen type (h and i); *Trifolium*‐type pollen aggregate at different focus (j and k); fern spore (l). The scale bar indicates 25 μm.

A huge amount of starch granules (exactly 19.065) was found in all samples from Pila cave, in a good state of preservation (Appendix S2‐Table S2). They were ascribable to Morphotype I and II, as showing the same morphological identifying characters previously described in the section of La Sassa cave and Table 1. Among all, 898 were attributed to Morphotype I (Figure 3a), 16.980 to Morphotype II (Figure 3b,c) and 1.187 were indicated as not determined, lacking diagnostic features.

#### 3.2.2 Pollen and spores

All samples from Pila cave showed the presence of pollen (Appendix S2‐Table S2). In total, 598 pollen grains and 2 spores were found and described in Table 1. Unfortunately, 82 pollen grains were not determined.

Five hundred and eleven ancient palynomorphs were classified as some of the pollen types already documented for La Sassa cave; therefore, the morphologies are those reported in detail in Table 1. In one calculus, a pollen was generically attributed to Betulaceae pollen (Sub Family Coryloideae, *Ostrya carpinifolia* or Sub Family Betuloideae, *Betula* sp.) (Figure 3d). Thirty‐eight samples out of 39 showed 492 palynomorphs with features comparable to Fagaceae pollen (e.g., *Castanea* or *Quercus* deciduous types) (Figure 3e). Eleven calculi contained a total of 15 pollen ascribable to Cupressaceae species (Figure 3f), while in the other three samples the presence of 3 microremains which, respectively, recalled pollen from subfamily Tilioideae, *Tilia* sp. (Figure 3g), Oleaceae and Poaceae species were registered.

The pollen types not shared with La Sassa cave were five. Two of them were ascribed to the Asteraceae family. In detail, the first one could not be identified at a lower taxonomic level, as not well‐conserved, although it showed shape and dimensions recalling the *Aster*‐type pollen (Figure 3h). The second one appeared similar to the pollen morphology of different genera from the Tribe Cichorieae (Subfamily Cichorioideae; e.g., *Taraxacum* sp., *Lactuca* sp., *Crepis* sp., *Hieracium* sp.) (Figure 3i). In one sample, an ancient aggregate of 4 pollen grains was found. These palynomorphs were attributed to *Trifolium*‐type (Fabaceae) (Koçyiğit et al., 2013) (Figure 3j,k). One microremain was generally linked to the Fabaceae pollen type, while another one was recognised as an Ulmaceae (e.g., *Ulmus* sp.) palynomorph.

Lastly, the microscopic analysis revealed two Pteridophytes‐monolete spores. One remained unidentified, while the other one (Figure 3l) was traced back to Polypodiaceae J. Presl & C. Presl family (more specifically *Polypodium* L.) (Adojoh et al., 2019; Daniau et al., 2019).

#### 3.2.3 Non‐pollen palynomorphs (NPPs)

**FIGURE 4:**
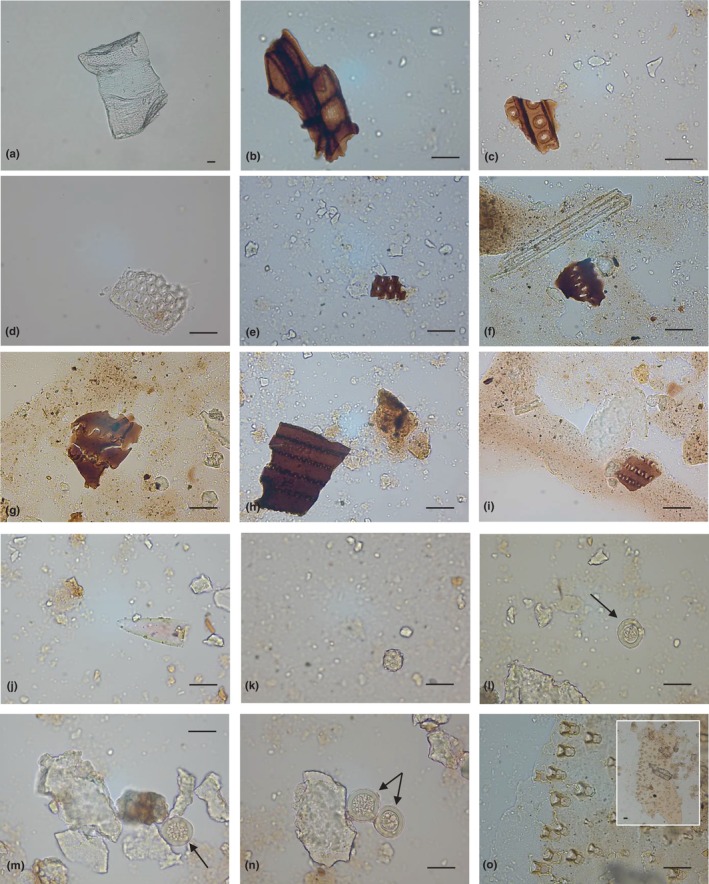
Mosaic selection of microdebris recovered from dental calculus samples of Pila cave. Examples of microremains captured by optic microscopy are shown. Fragments of plant vascular tissue (a and b); tracheid fragment in radial section from conifer wood, with uniseriate torus‐margo pits (c); microdebris consisting in tracheid pitting more seriate and alternate arrangement in radial walls (d and e); fragments of plant tissue containing articulate phytoliths (f–i); acute bulbous phytolith (j); globular echinate phytolith (k); parasite eggs indicated with arrows (l–n); magnification of a section of Lepidoptera wing (o), in the subpanel the whole microparticle. The scale bar indicates 30 μm.

**FIGURE 5:**
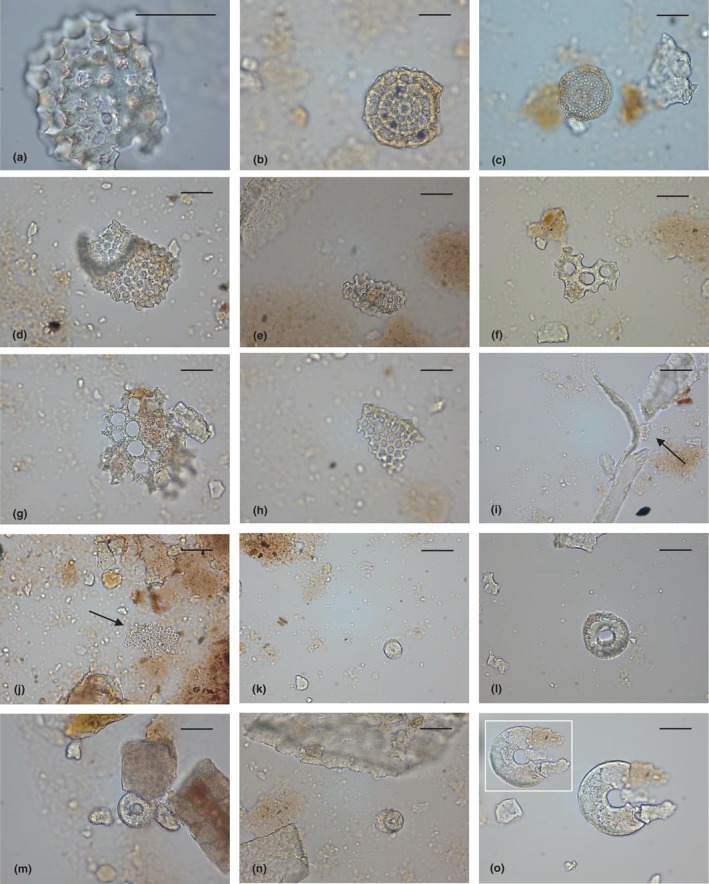
Siliceous microdebris retrieved from human calculi of Pila cave. Some of the images obtained by light microscopy analysis were shown. Microremains attributable to: Spumellaria order, putative Actinommidae family (a); Spumellaria order, putative Coccodiscidae family (b); putative Nassellaria radiolarians or diatoms (c–j); dissolved diatom frustules (k–n); putative amoeba shell at different focuses (o). Arrows localise the debris in panels (i and j). The scale bar measures 30 μm.

This systemic grouping consists of remains of organisms, often strictly reflecting local ecological conditions, which provide evidence about environment and human impact. Dental calculi from Pila cave show a great variety of NPPs from different kingdoms of life. Results were presented in sections and summarised in Appendix S2‐Table S2. Criteria and reliability of identification are reported below for each category, including palynodebris elements, animal micro debris, remains of algae or radiolarians and testate amoebae shells. The majority of the microremains were well preserved (Figures 4 and 5).

##### Palynodebris elements

In this section, 35 microdebris were classified according to Richter et al. (2004) and the International Code for Phytolith Nomenclature 2.0 (ICPN 2.0) proposed by the ICPT (International Committee for Phytolith Taxonomy) (Neumann et al., 2019). These elements included vessel elements with simple perforations (Figure 4a), portions of plant tissues (Figure 4b), phytoliths (i.e., amorphous silica particles forming in plant cells) and wood fragments. The latter were tracheid radial fragments from coniferous wood (earlywood), consisting of uniseriate lines of bordered pits (torus‐margo pits) (Figure 4c) and more‐seriate tracheid pits in radial sections with arrangement alternate (Figure 4d,e). Other microremains were identified as charred material (not completely carbonised), made up of sinuate long cells (Figure 4f–i), acute bulbous (Figure 4j) and globular echinate phytoliths (Figure 4k).

##### Animal micro debris

This section presents the morphological details of 59 non‐plant microremains found in calculi from Pila cave (Appendix S2‐Table S2).

Fifty‐eight parasite eggs, isolated from each other, were found in the sample PC 12. Their shape is well appreciable in Figure 4l–n. These eggs were round or slightly oval (average size: 32 μm in length; 28 μm in width) and the space between the membranes appeared generally smooth. This morphology commonly occurs in some cestode (tapeworms) genera of the order Cyclophyllidea, such as *Hymenolepis* and *Dipylidium*, infecting humans and domesticated animals (Garcia et al., 2018). As the appearance of the eggs produced by these tapeworms is similar and considering the state of preservation, we were not able to identify them at lower taxonomic ranks and discriminate their proper origin.

One tartar sample entrapped a fragment of invertebrate, which consisted mainly of an insect remain (Lepidoptera). It appeared still attached to small calculus particles and was recognised as part of the wing of a butterfly/moth with the attachment marks for the missing wing scales (Figure 4o) (Hardy et al., 2016; Henry, 2020; MacKenzie et al., 2021).

##### Diatoms and/or radiolarians

Fifty‐eight regularly perforated siliceous microfossils were tentatively attributed to fragments of the hole‐bearing globular shells Radiolaria and/or to the silica cell walls (frustules) of diatoms (microalgae) (Figure 5a–n), according to their size and pore patterns. Some of them could be recognised as the skeletons of Spumellaria radiolarians. Indeed, the morphology of this specific microremains recalled the spherical cortical shell of the Actinommidae family (Figure 5a) or the skeleton, with concentric chambered rings, from Coccodiscidae (e.g., *Coccodiscus* sp.) (Figure 5b). Other debris (and fragments) equipped with chequered or hexagonal pore arrangement, which was disposed of in both transverse and longitudinal rows, might be part of siliceous cone‐shaped skeletons from Nassellarians and/or centric diatom frustules (Figure 5c–j) (Gibaud et al., 2019; Matsuzaki & Itaki, 2019). Noteworthy, the morphology observed in the micro‐remains shown in Figure 5k–n, which would represent frustule cylindrical fragments and/or partially dissolved concentric ringleistes of *Aulacoseira* valves (more in general Aulacoseirales order) (diameter: 17–36 μm). Unfortunately, their perforation pattern appeared as not preserved. Finally, one calculus sample revealed a micro debris which resembled the pentagonal valve of the centric diatom genus *Triceratium* (e.g., Triceratiaceae), with characteristic ocelli in angles and inner raised pentagonal area.

##### Microdebris of doubtful origin

Two calculus samples revealed the presence of 2 microparticles which can be similar in appearance to autogenic/xenogenic tests of lobose testate amoebae. They appeared circular, with a single central opening in apertural view and a range of diameter equal to 33.4–76 μm (Figure 5o). A similar morphology usually occurs in Arcellinida species, such as *Arcella* or *Galeripora*, whose organisms inhabit marshes and other freshwater habitats and whose shells have been found to survive chemical treatments applied for pollen preparation methods (Andrews et al., 2021; González‐Miguéns et al., 2022; Payne et al., 2012). However, degraded valves of centric diatoms (e.g., *Hyalodiscus* species) cannot be excluded as the source of these peculiar microremains (Rott et al., 2009).

## 4 DISCUSSION

Our knowledge about ancient plant biodiversity and its exploitation by prehistorical communities still needs to be unravelled. In the present study, the microscopic analysis of human dental calculi from two Italian burial caves returned a high number of microfossils, including starches, different pollen types, pteridophyte spores and non‐pollen palynomorphs. These debris, considered as environmental indicators, allowed us to obtain some information about the phytoecological contexts in which the studied inhumates were framed. Deposition and preservation of organic matter in archaeological sediments are very complex phenomena linked to taphonomic processes, which influence the survival of plant macro remains. For this reason, the hidden evidence in dental calculus constitutes a precious undisturbed element for supposing the role of plants in ancient contexts.

Problematic is the attribution of starches (Morphotype I) to *Triticum* or *Hordeum* genera, due to their similar morphology and size. However, it is possible that these plants existed simultaneously, as macro botanicals attributable to different cereals, such as *Triticum monococcum* L., *T. dicoccum* Schübl., *T. aestivum*/*durum* and *Hordeum vulgare* L., have been found in Italian Neolithic, Copper and Bronze Age contexts, testifying cultivation and notable employment of these crops (Fiorentino et al., 2013; Rottoli & Castiglioni, 2009; Ucchesu et al., 2018). The record registered in the present contribution was also in line with other studies carried out on dental calculus relative to the same time span in Central Italy (D'Agostino et al., 2021; Lippi et al., 2017; Modi et al., 2021; Nava et al., 2021).

Particular attention must be paid to Panicoideae starches (Morphotype II), indicators of warm growing season. Despite the increasing evidence in prehistorical contexts, the distribution of these species and their importance in human life (Varalli et al., 2022) are still poorly clear. Panicoids are warm‐season crops easily cultivable in geographical areas characterised by moist winters and meagre summer precipitation as fast‐growing organisms with high adaptability (Stevens et al., 2016). Among them, millets are rather rare in central and southern Italian Neolithic‐Bronze Age contexts and are mainly attributed to *Panicum* and *Setaria* (Primavera et al., 2017; Rottoli & Castiglioni, 2009). In addition, isotopic data from Central Italy have indicated prehistorical people started to regularly consume panicoids only from BA (De Angelis et al., 2019; Romboni et al., 2023; Tafuri et al., 2009). The absence of this signal in previous and coeval sites (Cortese et al., 2022; De Angelis et al., 2019, 2021; Scorrano et al., 2019), even located in the same region, could be probably explained by low/random consumption of millets, not isotopically detectable. To date, no dietary investigation is available for Pila cave, while Romboni et al. (2023) have carried out isotopic analyses of carbon, nitrogen and strontium from the human bones recovered at La Sassa cave. The isotopic values have shown that different communities could have exploited the cave as a funerary shelter from CA to MBA, sometimes for selected individuals. Unfortunately, no settlement areas for those human groups have been discovered and the researchers have not determined the exact geographic origin of the individuals, as their isotopic signatures matched those of wide areas around the cave. Moreover, as no CA and EBA settlement has been ever registered in Central Italy with a such long presence as that of La Sassa cave (Romboni et al., 2023), it is possible to hypothesise a continuous attendance of the area for the studied inhabitants. Thus, we decided to extend our discussion to Central Italy and not uniquely to a regional level. For CA and EBA individuals from La Sassa, scholars have proposed a subsistence strategy grounded on terrestrial C_3_‐based resources, while for just one individual dated to MBA2 a diet based on C_4_ plants cannot be excluded. Nevertheless, as stated in Romboni et al. (2023), no isotopic value for C_4_ plants in Italian prehistory is available; thus, they have not had the possibility to check whether the recorded value for that single individual was derived reliably from an exclusive consumption of those species. Studies on dental calculus, instead, have testified knowledge of panicoids since the Late Palaeolithic in Southern Italy and Sicily (Carra et al., 2022), the Upper Palaeolithic in Abruzzi (Nava et al., 2021) and the Neolithic‐Bronze Age in Tuscany and Lazio (D'Agostino et al., 2021; D'Agostino, Di Marco, Marvelli, Marchesini, Rizzoli, et al., 2022; Lippi et al., 2017), even if the employment was a rare occasion of use. Evidence of grasses belonging to the Panicoideae subfamily was found in our samples, confirming their use and suggesting ecological aspects of the places frequented by the individuals. Indeed, the Paniceae species prefer mild climates and spreads near waterways and in coastal areas and may be considered suitable crops for challenging environments. Our results revealed that the individuals buried in La Sassa and Pila caves used C_4_ plants during CA‐BA. These starches may be attributed to grasses (e.g., *Setaria* sp., *Panicum* sp. and/or *Echinochloa* sp. P. Beauv.), whose consumption could also be supported by the detection of phytoliths, powerful tools to reconstruct past environments (Cabanes, 2020). Indeed, the latter, found in Pila cave samples, are indicative of monocots (e.g., sedge, reed), both domestic and wild (Goude et al., 2020), helping to define the herbaceous component of the landscape. In the current work, the analysis of starch granules retrieved from La Sassa samples showed the use of caryopses belonging to C_4_ plants in individuals dated to CA, EBA and MBA1/2. These data are not unexpected, as starches of similar morphology have already been found in Neolithic dental calculus from Grotta Continenza (Abruzzi; Nava et al., 2021) and Mora Cavorso (Lazio; D'Agostino, Di Marco, Marvelli, Marchesini, Rizzoli, et al., 2022) and CA‐BA samples from Grotta dello Scoglietto (Tuscany; Lippi et al., 2017) and Casale del Dolce (Lazio; D'Agostino et al., 2021), representing further proof of an already established presence of C_4_ plants in the human diet. Therefore, our data from La Sassa confirm the processing of Panicoideae in southern Lazio, already documented by D'Agostino et al. (2021), while those from Grotta Pila induce us to reflect on the presence and the exploitation of the same cereal resources even at higher altitudes and in the innermost areas of the region, even if only occasionally.

The failure to find legume starches in the investigated dental calculi seems to be in line with the evidence obtained from other studies on ancient tartar of prehistorical periods (Carra et al., 2022; D'Agostino, Di Marco, Marvelli, Marchesini, Rizzoli, et al., 2022; Lippi et al., 2017; Nava et al., 2021; Oxilia et al., 2021) and the scarce record of pulses registered in Italian Neolithic archaeological sites (Bouby et al., 2020; Celant, 2020), although Fabaceae are among the most adaptable plants to a wide range of habitats and dry legume seeds are capable of being well stored. However, pulses could have been less appreciated by the Neolithic people as a crop because of their low yield, compared to cereals and due to their content in a wide range of potentially toxic metabolites (e.g., saponins, alkaloids). The possibility that pulses were consumed in limited quantity, or only seasonally, needs to be also considered.

Finally, the discovery of brachysclereids in two samples from La Sassa cave appears interesting. This evidence suggested, for instance, the possible existence of Rosaceae plants in this area of Central Italy. Thus, the putative consumption, even occasional but shared among the individuals of the communities, of wild edible false fruits/fruits, such as pear and hawthorn, might be hypothesised. In support of this practice, the discovery of *Ficus* sp. and Rosaceae ancient DNA in Neolithic dental calculi from Mora Cavorso (Lazio, Central Italy) can be mentioned (D'Agostino, Di Marco, Marvelli, Marchesini, Rizzoli, et al., 2022), together with the finding of carpological records of *Corylus avellana* L., *Cornus mas* L., *Crataegus* sp., *Malus sylvestris* Mill., *Pyrus* sp., *Prunus* sp., *Quercus* sp. L and *Vitis vinifera* ssp. *sylvestris* L. (Gismondi et al., 2016; Rottoli & Castiglioni, 2009; Ucchesu et al., 2018) in Italy since the Neolithic, although usually in small amounts. Pollen observed in the calculus samples of both archaeological sites evidenced a similar vegetation composition in the surroundings of the caves. This assumption was supported by the fact that strontium isotope analysis on the human bones from La Sassa has suggested this context as a burial place by different villages, perhaps spreading in a radius of a few kilometres (Romboni et al., 2023). Thus, based on our results, the individuals were probably able to move in a mixed forest, characterised by deciduous (*Quercus*, Ulmaceae, Betulaceae as *Ostrya carpinifolia*), riparian (e.g., *Alnus*) and xerophilous evergreen (*Quercus*, Oleaceae) elements typical of Mediterranean regions characterised by mild winters and dry summers, inhaling pollen. This picture is confirmed by several pollen records from Lazio, in which a change in phytoassociations and a more warm‐temperate climate were established (Valle di Castiglione: 3480 ± 50 years B.P. Follieri et al., 1986; Lago Lungo: 3680 ± 70 years B.P. Calderoni et al., 1994; Mora Cavorso: 3640–3385 cal. years B.P. D'Agostino, Di Marco, Marvelli, Marchesini, Martínez‐Labarga, et al., 2022). As regards *Tilia* pollen, several pathways of inclusion in calculus can be considered: (i) accidental aspiration; (ii) use of decoctions of floral parts (dried inflorescences are traditional remedies against cough, cold and bronchitis; Oniszczuk & Podgórski, 2015; Sroka & Bełz, 2009; and references within); (iii) use of a fermented honey‐based drink, pure honey, or beehive product (e.g., honeydew) (Carboni et al., 2015; D'Agostino et al., 2021). This latter hypothesis can be assumed also for the numerous Fagaceae pollen (e.g., *Quercus* deciduous group) identified in Pila samples and to a lesser extent in La Sassa specimens. Pollen data, partially outlining the ancient plant diversity, suggested that the environs of the caves could include: (i) cosmopolitan non‐arboreal taxa generally indicative of open environments (e.g., Poaceae, Asteraceae, *Aster‐*type in mountain steppe zone); (ii) important pastureland or grazing indicators (e.g., Cichorieae); (iii) arboreal taxa related to local edaphic requirements (e.g., Cupressaceae) and not indicative of specific ecological conditions (e.g., Fabaceae undetermined).

Individuals from both caves showed Pteridophyte elements, uncommon microparticles rarely observed in ancient human dental calculi (D'Agostino et al., 2021; Fiorin et al., 2019). Fern spores are resistant to unfavourable environmental conditions; thus, this finding could derive from their sedimentation on food or from the activities performed in poorly drained habitats (e.g., swamps, areas near rivers and lakes, coastal plain landscapes). Fragments of gymnosperm wood (e.g., *Pinus*), charred material (not completely carbonised) with phytoliths and an insect remain from calculi of Pila cave indicated the presence of taxa with different ecological and climatic requirements. Dental calculus has been proved to preserve such types of microparticles, which could have different pathways of inclusions (Hardy et al., 2017; Nava et al., 2021; Radini et al., 2016). In particular, insects are common food contaminants ubiquitous in soil and air, while wood debris can enter the mouth by toothpick use, or through not deliberate ingestion linked to working practices involving fire or environmental (domestic) pollution (Cristiani et al., 2016; Hardy et al., 2016; Juhola et al., 2019; MacKenzie et al., 2021; Radini et al., 2016, 2017). However, we also suggest they can derive from poorly washed food still preserving traces of soil.

Parasitic particles from archaeo‐anthropological materials denounce a status of zoonosis, whose existence is established starting from Neolithic domestication events and sharing of living areas (Mazoyer & Roudart, 2006). Worldwide reports of helminth eggs have been reported for populations of Fertile Crescent (Diamond, 2002; Driscoll et al., 2009; Zeder, 2008), Western Iran (Paknezhad et al., 2017) and Atacama Desert (northern Chile) (Ramirez et al., 2021). The discovery of several cestode eggs in the dental calculus of an individual buried in the Pila cave could seem counterintuitive. However, accidental infection of the most prevalent human cestodiasis in the world (Acha & Szyfres, 2003) can easily be acquired by touching the mouth with contaminated fingers and/or ingesting food, soil or water contaminated with faeces by companion animals or parasitic insects (Garcia et al., 2018) and/or presence of dung (animal and/or human) in the living areas. Similar evidence has already been registered for dental calculus samples (Juhola et al., 2019); thus, the recovery of cestode eggs from tartar would seem to be sufficient for the establishment of a parasite's existence of public health significance (as a disease agent) among the prehistorical individuals buried in Pila cave. From length‐width measurements and morphological features, the eggs we detected could correspond to *Hymenolepis* spp. and/or *Dipylidium caninum*. Unfortunately, a deeper classification of the tapeworm's eggs is not possible based only on morphology and size (Garcia et al., 2018). It is important to underline that no parasite was found in the control washings both before and after the decontamination procedure.

The inclusion of non‐dietary microfossils in dental calculus samples, such as silica‐rich fragments, has already been documented in the literature. The recovery of such type of remains, including microalgae, provided evidence of the phytoplancton diversity from water sources in archaeological contexts, together with demographic implications of settlement strategy and geographic mobility (Dudgeon & Tromp, 2014; King et al., 2017; Stone & Yost, 2020). Among many, Radiolarians and diatoms possess biogenic silica structures, which can be resilient to alteration by salivary digestion, masticatory activities and acids. Radiolarians are mainly seawater protozoan, whose ecological niches are rather complex and whose species may vary based on season, depth and/or nutritional availability. Diatoms are ubiquitous, unicellular microalgae mostly colonising aquatic ecosystems, both as planktonic forms (suspended in the water column) and as benthic, surface‐attached organisms. As photosynthetic microorganisms, diatoms proliferate in sunlit surface waters of ponds and streams, as well as in brackish and marine systems, but they can also thrive in terrestrial habitats such as moist soil and wet rocks. Thus, all these organisms may represent proxies of brackish, marine and fresh‐water sources or of the mixing of seawater and fresh water (e.g., estuaries or fossil brackish water aquifers) (Adojoh et al., 2019; Smol & Stoermer, 2010; Suzuki & Not, 2015). In our case, some siliceous microparticles, different in pore size and arrangement, could be tentatively attributed to both the globular perforated skeletons of Radiolaria and to the silica cell walls (frustules) of centric diatoms, although most of them appeared highly fragmented. It would be very interesting to understand if their fragmentation preceded the entrapment in calculus deposits. The hypotheses for their presence in dental calculus may be the consumption of: (i) local drinking water (also ephemeral rainwater collected in vessels); (ii) local aquatic food organisms (e.g., fish, shellfish, macroalgae); (iii) dried fish, macroalgae or other seafood collected before reaching the settlement areas neighbouring to Pila cave (e.g., travel to the coast); (iv) mineral concretions (e.g., salt crystals) in which fragments of diatoms may have precipitated. Finally, microparticles referred to as ‘of doubtful origin’ and tentatively attributable to lobose testate amoebae shells and/or degraded diatom frustules, could testify to a wide variety of aquatic and terrestrial environments: lake and river sediments, peat bogs, coastal environments, glaciers and dry mosses growing on different substrates (Andrews et al., 2021; Payne et al., 2012).

## 5 CONCLUSION

Beyond macrobotanicals, which represent direct evidence of the existence of plant species in the past, recently, many research groups have taken advantage of the analysis of plant microremains retrieved from ancient dental calculus, to open a window on the palaeoecological conditions and past plant diversity. The development and the distribution of phyto‐associations, which are influenced by geographic and climatic variability, certainly have affected the subsistence strategies of prehistoric communities. To date, unfortunately, little is known about how our ancestors selected plant species in nature and exploited their diversity. Thus, our work demonstrated that dental calculus analysis can be used to extrapolate information about human‐plant interaction and to gain a glimpse on the past landscape, corroborating and even expanding the insights already obtained by palynological investigations. Here, we also proved the survival in the dental calculus of a wide range of material items that may reflect ecological/environmental details of a specific context.

The identification of starches, pollen and non‐pollen palynomorphs was able to reveal the plant resources that came into contact with pre‐ and proto‐historical communities from Central Italy. For instance, the exploitation of C_4_ plants, a debated research topic. Indeed, we highlighted that panicoids have started to be consumed and/or processed from the individuals living in the areas around La Sassa and Pila caves, already from the Copper Age, even if only for brief periods. These plant species, possessing a very short lifecycle, may have been able to sprout and grow locally outside of the usual season, maybe thanks to more favourable climatic conditions.

The whole starch documentation suggested the existence of both C_3_ and C_4_ grasses, even though it couldn't clarify the contribution of gathered wild plants and domesticated cultivars. On the other hand, the detection of other plant microparticles, considered as environmental multiproxy (e.g., diatoms, pollen, brachysclereids), helped us to infer past contexts, characterised by both water and terrestrial plant species. Our evidence for the consumption of at least two different starchy plant taxa, in addition to the direct proofs for the consumption of wild edible fruits, maybe honey and the use of plant‐based raw materials, suggests that the individuals had a detailed knowledge of the phytoecological context of their settlement areas, including the relative biodiversity.

It is well known that the same cultural phase shows time discrepancies in various geographical districts, although both our data sets indicate homogeneity in the vegetation for the areas frequented by the individuals. Thermophilous taxa and evergreen elements retrieved from dental calculi suggested that during the Copper and Middle Bronze Age mixed coniferous–deciduous woodlands existed in Central Italy.

## AUTHOR CONTRIBUTIONS

**Alessia D'Agostino:** Conceptualization (equal); data curation (equal); formal analysis (equal); investigation (equal); methodology (equal); validation (equal); writing – original draft (equal); writing – review and editing (equal). **Gabriele Di Marco:** Investigation (equal); writing – review and editing (equal). **Mario Federico Rolfo:** Conceptualization (equal); resources (equal); validation (equal); writing – review and editing (equal). **Luca Alessandri:** Conceptualization (equal); resources (equal); writing – review and editing (equal). **Silvia Marvelli:** Validation (equal); writing – review and editing (equal). **Roberto Braglia:** Validation (equal); writing – review and editing (equal). **Roberta Congestri:** Validation (equal); writing – review and editing (equal). **Federica Berrilli:** Validation (equal); writing – review and editing (equal). **Maria Felicita Fuciarelli:** Validation (equal); writing – review and editing (equal). **Angelica Ferracci:** Resources (equal); writing – review and editing (equal). **Antonella Canini:** Funding acquisition (equal); resources (equal); writing – review and editing (equal). **Angelo Gismondi:** Conceptualization (lead); data curation (equal); funding acquisition (equal); methodology (equal); resources (equal); validation (equal); writing – original draft (lead); writing – review and editing (equal).

## CONFLICT OF INTEREST STATEMENT

The authors declare no conflicts of interest.

## Supporting information


Appendix S1

Appendix S2

## Data Availability

All the data described in the manuscript are reported in the text or the supplemental material files.

## References

[ece311053-bib-0001] Acha, P. N. , & Szyfres, B. (2003). Hymenolepiasis. In O. O. Barriga (Ed.), Zoonoses and communicable diseases common to man and animals (3rd ed., pp. 199–204). Pan American Health Organization.

[ece311053-bib-0002] Adojoh, O. , Fabienne, M. , Duller, R. , & Osterloff, P. (2019). Taxonomy and phytoecology of palynomorphs and non‐pollen palynomorphs: A refined compendium from the West Africa Margin. Biodiversity International Journal, 3(5), 188–200.

[ece311053-bib-0003] Ahituv, H. , & Henry, A. G. (2022). An initial key of starch grains from edible plants of the Eastern Mediterranean for use in identifying archaeological starches. Journal of Archaeological Science: Reports, 42, 103396.

[ece311053-bib-0004] Alessandri, L. , Cardello, G. L. , Attema, P. A. J. , Baiocchi, V. , De Angelis, F. , Del Pizzo, S. , Di Ciaccio, F. , Fiorillo, A. , Gatta, M. , Monti, F. , Onori, M. , Rolfo, M. F. , Romboni, M. , Sottili, G. , & Troisi, S. (2021). Reconstructing the Late Pleistocene–Anthropocene interaction between the neotectonic and archaeological landscape evolution in the Apennines (La Sassa cave, Italy). Quaternary Science Reviews, 265, 107067.

[ece311053-bib-0005] Andrews, L. O. , Payne, R. J. , & Swindles, G. T. (2021). Testate amoebae as non‐pollen palynomorphs in pollen slides: Usefulness and application in palaeoenvironmental reconstruction. Geological Society ‐ Special Publications, 511(1), 151–158.

[ece311053-bib-0006] Barclay, G. (2007). Plant anatomy. In K. Roberts (Ed.), Handbook of plant science (pp. 13–26). Wiley.

[ece311053-bib-0007] Bellotti, P. , Calderoni, G. , Dall'Aglio, P. L. , D'Amico, C. , Davoli, L. , Di Bella, L. , D'Orefice, M. , Esu, D. , Ferrari, K. , Mazzanti, M. B. , Mercuri, A. M. , Tarragoni, C. , & Torri, P. (2016). Middle‐to late‐Holocene environmental changes in the Garigliano delta plain (Central Italy): Which landscape witnessed the development of the Minturnae Roman colony? Holocene, 26, 1457–1471.

[ece311053-bib-0008] Berglund, B. E. , & Ralska‐Jasiewiczowa, M. (1986). Pollen analysis and pollen diagrams. In B. E. Berglund (Ed.), Handbook of Holocene palaeoecology and palaeohydrology (pp. 455–484). Wiley.

[ece311053-bib-0009] Bouby, L. , Marinval, P. , Durand, F. , Figueiral, I. , Briois, F. , Martzluff, M. , Perrin, T. , Valdeyron, N. , Vaquer, J. , Guilaine, J. , & Manen, C. (2020). Early Neolithic (ca. 5850‐4500 cal BC) agricultural diffusion in the Western Mediterranean: An update of archaeobotanical data in SW France. PLoS One, 15(4), e0230731. 32240184 10.1371/journal.pone.0230731 PMC7117749

[ece311053-bib-0010] Butterworth, P. J. , Warren, F. J. , & Ellis, P. R. (2011). Human α‐amylase and starch digestion: An interesting marriage. Starch‐Stärke, 63, 395–405.

[ece311053-bib-0011] Cabanes, D. (2020). Phytolith analysis in paleoecology and archaeology. In A. G. Henry (Ed.), Handbook for the analysis of micro‐particles in archaeological samples (pp. 255–288). Springer.

[ece311053-bib-0012] Calderoni, G. , Carrara, C. , Ferreli, L. , Follieri, M. , Gliozzi, E. , Magri, D. , Narcisi, B. , Parotto, M. , Sadori, L. , & Serva, L. (1994). Palaeoenvironmental, palaeoclimatic and chronological interpretations of a late Quaternary sediment core from Piana di Rieti (central Apennines, Italy). Giornale di Geologia, 56(2), 43–72.

[ece311053-bib-0013] Carboni, G. , Celant, A. , Forte, V. , Magri, D. , Nunziante Cesaro, S. , & Anzidei, A. P. (2015). Inebriarsi per l'aldilà: Bevande alcoliche nelle necropoli di facies Rinaldone e Gaudo dell'area romana. In *Atti della L Riunione Scientifica dell'Istituto Italiano di Preistoria e Protostoria*.

[ece311053-bib-0014] Carra, M. , Zupancich, A. , Fiorin, E. , Sarti, L. , Vetro, D. L. , Martini, F. , & Cristiani, E. (2022). Plant foods in the Late Palaeolithic of Southern Italy and Sicily: Integrating carpological and dental calculus evidence. Quaternary International, 653–654, 53–68.

[ece311053-bib-0015] Celant, A. (2020). Indagini paleobotaniche su macroresti vegetali dai siti neo‐eneolitici del territorio di Roma. In A. P. Anzidei & C. Carboni (Eds.), Roma prima del mito. Abitati e necropoli dal Neolitico alla prima età dei Metalli nel territorio di Roma (VI‐III millennio a.C.) (pp. 687–704). Archaeopress Publishing Ltd.

[ece311053-bib-0016] Cortese, F. , De Angelis, F. , Achino, K. F. , Bontempo, L. , di Cicco, M. R. , Gatta, M. , Lubritto, C. , Salari, L. , Silvestri, L. , Rickards, O. , & Rolfo, M. F. (2022). Isotopic reconstruction of the subsistence strategy for a Central Italian Bronze Age community (Pastena cave, 2nd millennium BCE). Archaeological and Anthropological Sciences, 14(10), 1–14.

[ece311053-bib-0017] Cristiani, E. , Radini, A. , Edinborough, M. , & Borić, D. (2016). Dental calculus reveals Mesolithic foragers in the Balkans consumed domesticated plant foods. PNAS, 113(37), 10298–10303. 10.1073/pnas.1603477113 27573829 PMC5027412

[ece311053-bib-0018] Cristiani, E. , Radini, A. , Zupancich, A. , Gismondi, A. , D'Agostino, A. , Ottoni, C. , Carra, M. , Vukojičić, S. , Constantinescu, M. , Antonović, D. , Price, T. D. , & Borić, D. (2021). Wild cereal grain consumption among Early Holocene foragers of the Balkans predates the arrival of agriculture. eLife, 10, e72976. 34850680 10.7554/eLife.72976 PMC8782571

[ece311053-bib-0019] D'Agostino, A. , Canini, A. , Di Marco, G. , Nigro, L. , Spagnoli, F. , & Gismondi, A. (2020). Investigating plant micro‐remains embedded in dental calculus of the Phoenician inhabitants of Motya (Sicily, Italy). Plants, 9(10), 1395. 33092237 10.3390/plants9101395 PMC7590007

[ece311053-bib-0020] D'Agostino, A. , Di Marco, G. , Marvelli, S. , Marchesini, M. , Martínez‐Labarga, J. M. , Rolfo, M. F. , Canini, A. , & Gismondi, A. (2022). Pollen record of the Late Pleistocene–Holocene stratigraphic sequence and current plant biodiversity from Grotta Mora Cavorso (Simbruini Mountains, Central Italy). Ecology and Evolution, 12, e9486. 10.1002/ece3.9486 36381401 PMC9643123

[ece311053-bib-0021] D'Agostino, A. , Di Marco, G. , Marvelli, S. , Marchesini, M. , Rizzoli, E. , Rolfo, M. F. , Canini, A. , & Gismondi, A. (2022). From the inside out: Neolithic dental calculi returned environmental proxies and evidence for consumption of wild edible fruits and herbs. Communications Biology, 5, 1384. 36536113 10.1038/s42003-022-04354-0 PMC9763411

[ece311053-bib-0022] D'Agostino, A. , Di Marco, G. , Rubini, M. , Marvelli, S. , Rizzoli, E. , Canini, A. , & Gismondi, A. (2021). Environmental implications and evidence of natural products from dental calculi of a Neolithic–Chalcolithic community (central Italy). Scientific Reports, 11(1), 1–13. 34021220 10.1038/s41598-021-89999-3 PMC8140145

[ece311053-bib-0023] Daniau, A. L. , Desprat, S. , Aleman, J. C. , Bremond, L. , Davis, B. , Fletcher, W. , Marlon, J. R. , Marquer, L. , Montade, V. , Morales‐Molino, C. , Naughton, F. , Rius, D. , & Urrego, D. H. (2019). Terrestrial plant microfossils in palaeoenvironmental studies, pollen, microcharcoal and phytolith. Towards a comprehensive understanding of vegetation, fire and climate changes over the past one million years. Revue de Micropaleontologie, 63, 1–35.

[ece311053-bib-0024] De Angelis, F. , Pellegrini, M. , Martínez‐Labarga, C. , Anzivino, L. , Scorrano, G. , Brilli, M. , Giustini, F. , Angle, M. , Calattini, M. , Carboni, G. , Catalano, P. , Ceccaroni, E. , Cosentino, S. , Di Giannantonio, S. , Isola, I. , Martini, F. , Pacciani, E. , Radina, F. , Rolfo, M. F. , … Rickards, O. (2021). Exploring mobility in Italian Neolithic and Copper Age communities. Scientific Reports, 11, 2697. 33514802 10.1038/s41598-021-81656-z PMC7846752

[ece311053-bib-0025] De Angelis, F. , Scorrano, G. , Martínez‐Labarga, C. , Giustini, F. , Brilli, M. , Pacciani, E. , Silvestrini, M. , Calattini, M. , Volante, N. , Martini, F. , Sarti, L. , & Rickards, O. (2019). Eneolithic subsistence economy in Central Italy: First dietary reconstructions through stable isotopes. Archaeological and Anthropological Sciences, 2, 1–16.

[ece311053-bib-0026] Denk, T. , & Tekleva, M. V. (2014). Pollen morphology and ultrastructure of *Quercus* with focus on Group Ilex (= *Quercus* Subgenus *Heterobalanus* (Oerst.) Menitsky): Implications for oak systematics and evolution. Grana, 53, 255–282.

[ece311053-bib-0027] Di Rita, F. , Celant, A. , & Magri, D. (2010). Holocene environmental instability in the wetland north of the Tiber delta (Rome, Italy): Sea‐lake man interactions. Journal of Paleolimnology, 44, 51–67.

[ece311053-bib-0028] Di Rita, F. , Lirer, F. , Bonomo, S. , Cascella, A. , Ferraro, L. , Florindo, F. , Insinga, D. D. , Lurcock, P. C. , Margaritelli, G. , Petrosino, P. , Rettori, R. , Vallefuoco, M. , & Magri, D. (2018). Late Holocene forest dynamics in the Gulf of Gaeta (Central Mediterranean) in relation to NAO variability and human impact. Quaternary Science Reviews, 179, 137–141.

[ece311053-bib-0029] Diamond, J. (2002). Evolution, consequences and future of plant and animal domestication. Nature, 418(6898), 700–707. 12167878 10.1038/nature01019

[ece311053-bib-0030] Doorenbosch, M. , & Field, M. H. (2019). A Bronze Age palaeoenvironmental reconstruction from the Fondi basin, southern Lazio, central Italy. Quaternary International, 499, 221–230.

[ece311053-bib-0031] Driscoll, C. A. , Macdonald, D. W. , & O'Brien, S. J. (2009). From wild animals to domestic pets, an evolutionary view of domestication. Proceedings of the National Academy of Sciences of the United States of America, 106, 9971–9978. 10.1073/pnas.0901586106 19528637 PMC2702791

[ece311053-bib-0032] Dudgeon, J. V. , & Tromp, M. (2014). Diet, geography and drinking water in Polynesia: Microfossil research from archaeological human dental calculus, Rapa Nui (Easter Island). International Journal of Osteoarchaeology, 24(5), 634–648.

[ece311053-bib-0033] Erdtman, G. (1986). Pollen morphology and plant taxonomy: Angiosperms. Brill Archive.

[ece311053-bib-0034] Faegri, K. , & Iversen, J. (1989). In K. Faegri , P. E. Kaland , & K. Krzywinski (Eds.), Textbook of pollen analysis (4th ed.). John Wiley and Sons.

[ece311053-bib-0035] Farrer, A. G. , Wright, S. L. , Skelly, E. , Eisenhofer, R. , Dobney, K. , & Weyrich, L. S. (2021). Effectiveness of decontamination protocols when analyzing ancient DNA preserved in dental calculus. Scientific Reports, 11(1), 7456. 33811235 10.1038/s41598-021-86100-w PMC8018977

[ece311053-bib-0036] Fiorentino, G. , Caldara, M. , De Santis, V. , D'Oronzo, C. , Muntoni, I. M. , Simone, O. , Primavera, M. , & Radina, F. (2013). Climate changes and human–environment interactions in the Apulia region of southeastern Italy during the Neolithic period. Holocene, 23(9), 1297–1316.

[ece311053-bib-0037] Fiorin, E. , Sáez, L. , & Malgosa, A. (2019). Ferns as healing plants in medieval Mallorca, Spain? Evidence from human dental calculus. International Journal of Osteoarchaeology, 29, 82–90.

[ece311053-bib-0038] Follieri, M. , Magri, D. , & Sadori, L. (1986). Pollen analysis. 14C dating, geochemical features, faunistic and pollen analyses of the uppermost 10 m core from Valle di Castiglione (Rome, Italy). Geologica Romana, 10, 287–308.

[ece311053-bib-0039] Garcia, L. S. , Arrowood, M. , Kokoskin, E. , Paltridge, G. P. , Pillai, D. R. , Procop, G. W. , Ryan, N. , Shimizu, R. Y. , & Visvesvara, G. (2018). Practical guidance for clinical microbiology laboratories: Laboratory diagnosis of parasites from the gastrointestinal tract. Clinical Microbiology Reviews, 31(1), e00025‐17. 29142079 10.1128/CMR.00025-17 PMC5740970

[ece311053-bib-0040] Gibaud, A. , Villanova, J. , Cherkas, O. , Bulou, A. , Ouanssi, L. K. , Mcheik, A. , Cassaignon, S. , Lopez, P. J. , & Berthier, S. (2019). Analysis of diatoms by holotomography. Surfaces and Interfaces, 17, 100358.

[ece311053-bib-0041] Gismondi, A. , Di Marco, G. , Martini, F. , Sarti, L. , Crespan, M. , Martínez‐Labarga, C. , Rickards, O. , & Canini, A. (2016). Grapevine carpological remains revealed the existence of a Neolithic domesticated *Vitis vinifera* L. specimen containing ancient DNA partially preserved in modern ecotypes. Journal of Archaeological Science, 69, 75–84.

[ece311053-bib-0042] González‐Miguéns, R. , Soler‐Zamora, C. , Villar‐Depablo, M. , Todorov, M. , & Lara, E. (2022). Multiple convergences in the evolutionary history of the testate amoeba family Arcellidae (Amoebozoa: Arcellinida: Sphaerothecina): When the ecology rules the morphology. Zoological Journal of the Linnean Society, 194(4), 1044–1071.

[ece311053-bib-0043] Goude, G. , Salazar‐García, D. C. , Power, R. C. , Rivollat, M. , Gourichon, L. , Deguilloux, M. F. , Pemonge, M. H. , Bouby, L. , & Binder, D. (2020). New insights on Neolithic food and mobility patterns in Mediterranean coastal populations. American Journal of Physical Anthropology, 173(2), 218–235. 32557548 10.1002/ajpa.24089

[ece311053-bib-0044] Goude, G. , Salazar‐García, D. C. , Power, R. C. , Terrom, J. , Rivollat, M. , Deguilloux, M. F. , Pemonge, M. H. , le Bailly, M. , Andre, G. , Coutelas, A. , & Hauzeur, A. (2019). A multidisciplinary approach to Neolithic life reconstruction. Journal of Archaeological Method and Theory, 26(2), 537–560.

[ece311053-bib-0045] Grímsson, F. , Zetter, R. , Grimm, G. W. , Pedersen, G. K. , Pedersen, A. K. , & Denk, T. (2015). Fagaceae pollen from the early Cenozoic of West Greenland: Revisiting Engler's and Chaney's Arcto‐Tertiary hypotheses. Plant Systematics and Evolution, 301, 809–832. 25620836 10.1007/s00606-014-1118-5 PMC4299674

[ece311053-bib-0046] Halbritter, H. (2016). Ostrya carpinifolia . In PalDat ‐ A palynological database. Retrieved September 1, 2022, from https://www.paldat.org/pub/Ostrya_carpinifolia/302055

[ece311053-bib-0047] Halbritter, H. , Heigl, H. , & Auer, W. (2021). Tilia cordata . In PalDat ‐ A palynological database. Retrieved September 1, 2022, from https://www.paldat.org/pub/Tilia_cordata/306424

[ece311053-bib-0048] Hardy, K. , Buckley, S. , Collins, M. J. , Estalrrich, A. , Brothwell, D. , Copeland, L. , & Rosas, A. (2012). Neanderthal medics? Evidence for food, cooking, and medicinal plants entrapped in dental calculus. Naturwissenschaften, 99, 617–626. 22806252 10.1007/s00114-012-0942-0

[ece311053-bib-0049] Hardy, K. , Buckley, S. , & Copeland, L. (2018). Pleistocene dental calculus: Recovering information on Paleolithic food items, medicines, paleoenvironment and microbes. Evolutionary Anthropology, 27(5), 234–246. 30326183 10.1002/evan.21718

[ece311053-bib-0050] Hardy, K. , Radini, A. , Buckley, S. , Blasco, R. , Copeland, L. , Burjachs, F. , Girbal, J. , Yll, R. , Carbonell, E. , & Bermúdez de Castro, J. M. (2017). Diet and environment 1.2 million years ago revealed through analysis of dental calculus from Europe's oldest hominin at Sima del Elefante, Spain. The Science of Nature, 104(1), 1–5. 10.1007/s00114-016-1420-x 27981368

[ece311053-bib-0051] Hardy, K. , Radini, A. , Buckley, S. , Sarig, R. , Copeland, L. , Gopher, A. , & Barkai, R. (2016). Dental calculus reveals potential respiratory irritants and ingestion of essential plant‐based nutrients at Lower Palaeolithic Qesem Cave Israel. Quaternary International, 398, 129–135.

[ece311053-bib-0052] Henry, A. G. (Ed.). (2020). Other microparticles: Volcanic glass, minerals, insect remains, feathers, and other plant parts. In Handbook for the analysis of micro‐particles in archaeological samples (pp. 289–295). Springer.

[ece311053-bib-0053] Henry, A. G. , Brooks, A. S. , & Piperno, D. R. (2011). Microfossils in calculus demonstrate consumption of plants and cooked foods in Neanderthal diets (Shanidar III, Iraq; Spy I and II, Belgium). Proceedings of the National Academy of Sciences of the United States of America, 108(2), 486–491. 21187393 10.1073/pnas.1016868108 PMC3021051

[ece311053-bib-0054] ICSN . (2011). The international code for starch nomenclature . Retrieved March 9, 2022, from http://fossilfarm.org/ICSN/Code.html

[ece311053-bib-0055] ISO 3950 . (2009). Dentistry‐designation system for teeth and areas of the oral cavity. ISO. 9791262

[ece311053-bib-0056] Jin‐tan, Z. (1982). Study on the pollen morphology of the Chinese family Oleaceae. Journal of Integrative Plant Biology, 24(6), 499–505.

[ece311053-bib-0057] Juhola, T. , Henry, A. G. , Kirkinen, T. , Laakkonen, J. , & Väliranta, M. (2019). Phytoliths, parasites, fibers, and feathers from dental calculus and sediment from Iron Age Luistari cemetery, Finland. Quaternary Science Reviews, 222, 105888.

[ece311053-bib-0058] Khalik, K. A. , Maesen, L. J. G. , Kopman, W. J. M. , & Berg, R. G. (2002). Numerical taxonomic study of some tribes of Brassicaceae from Egypt. Plant Systematics and Evolution, 233, 207–221.

[ece311053-bib-0059] King, D. J. , Searcy, M. T. , Yost, C. L. , & Waller, K. (2017). Corn, beer, and marine resources at Casas Grandes, Mexico: An analysis of prehistoric diets using microfossils recovered from dental calculus. Journal of Archaeological Science: Reports, 16, 365–379.

[ece311053-bib-0060] Koçyiğit, M. , Keskin, M. , & Daştan, T. (2013). Pollen morphology of some *Trifolium* species, which are favorite plants of honeybees in Istanbul. Journal of the Faculty of Pharmacy of İstanbul Üniversity, 43, 85–94.

[ece311053-bib-0061] Lin, S. , Lin, D. , Wu, B. , Ma, S. , Sun, S. , Zhang, T. , Zhang, W. , Bai, Y. , Wang, Q. , & Wu, J. (2022). Morphological and developmental features of stone cells in *Eriobotrya* fruits. Frontiers in Plant Science, 13, 823993. 35154231 10.3389/fpls.2022.823993 PMC8828544

[ece311053-bib-0062] Lippi, M. M. , Pisaneschi, L. , Sarti, L. , Lari, M. , & Moggi‐Cecchi, J. (2017). Insights into the Copper‐Bronze Age diet in central Italy: Plant microremains in dental calculus from Grotta dello Scoglietto (Southern Tuscany, Italy). Journal of Archaeological Science: Reports, 15, 30–39.

[ece311053-bib-0063] MacKenzie, L. , Speller, C. F. , Holst, M. , Keefe, K. , & Radini, A. (2021). Dental calculus in the industrial age: Human dental calculus in the Post‐Medieval period, a case study from industrial Manchester. Quaternary International, 653–654, 114–126. 10.1016/j.quaint.2021.09.020 PMC10615834 37915533

[ece311053-bib-0064] Magri, D. , & Sadori, L. (1999). Late Pleistocene and Holocene pollen stratigraphy at Lago di Vico, central Italy. Vegetation History and Archaeobotany, 8, 247–260.

[ece311053-bib-0065] Mäkelä, E. M. (1996). Size distinctions between *Betula* pollen types—A review. Grana, 35(4), 248–256.

[ece311053-bib-0066] Matsuzaki, K. M. , & Itaki, T. (2019). Late Miocene polycystine radiolarians of the Japan Sea (IODP Exp. 346 Site U1425). Bulletin. Geological Survey of Japan, 70, 195–209.

[ece311053-bib-0067] Mazoyer, M. , & Roudart, L. (2006). A history of world agriculture: From the Neolithic age to the current crisis (pp. 71–101). NYU Press.

[ece311053-bib-0068] Mercuri, A. M. , Accorsi, C. A. , & Mazzanti, M. B. (2002). The long history of *Cannabis* and its cultivation by the Romans in central Italy, shown by pollen records from Lago Albano and Lago di Nemi. Vegetation History and Archaeobotany, 11, 263–276.

[ece311053-bib-0069] Mercuri, A. M. , & Sadori, L. (2012). Climate changes and human settlements since the Bronze age period in central Italy. Rendiconti Online della Società Geologica Italiana, 18, 32–34.

[ece311053-bib-0070] Modi, A. , Attolini, D. , Zaro, V. , Pisaneschi, L. , Innocenti, G. , Vai, S. , Caramelli, D. , Cecchi, J. M. , Quagliariello, A. , Lippi, M. M. , & Lari, M. (2021). Combined metagenomic and archaeobotanical analyses on human dental calculus: A cross‐section of lifestyle conditions in a Copper Age population of central Italy. Quaternary International, 653–654, 69–81.

[ece311053-bib-0071] Moore, P. D. , Webb, J. A. , & Collinson, M. E. (1991). Pollen analysis (2nd ed.). Blackwell Scientifc Publications.

[ece311053-bib-0072] Nava, A. , Fiorin, E. , Zupancich, A. , Carra, M. , Ottoni, C. , Di Carlo, G. , Vozza, I. , Brugnoletti, O. , Alhaique, F. , Cremonesi, R. G. , Coppa, A. , Bondioli, L. , Borić, D. , & Cristiani, E. (2021). Multipronged dental analyses reveal dietary differences in last foragers and first farmers at Grotta Continenza, central Italy (15,500–7000 BP). Scientific Reports, 11, 4261. 33608594 10.1038/s41598-021-82401-2 PMC7895915

[ece311053-bib-0073] Neumann, K. , Strömberg, C. A. E. , Ball, T. , Albert, R. M. , Vrydaghs, L. , & Cummings, L. S. (2019). International code for phytolith nomenclature (ICPN) 2.0. Annals of Botany, 124, 189–199. 31334810 10.1093/aob/mcz064 PMC6758648

[ece311053-bib-0074] Nikitkova, A. E. , Haase, E. M. , & Scannapieco, F. A. (2013). Taking the starch out of oral biofilm formation: Molecular basis and functional significance of salivary α‐amylase binding to oral streptococci. Applied and Environmental Microbiology, 79, 416–423. 23144140 10.1128/AEM.02581-12 PMC3553756

[ece311053-bib-0075] Norström, E. , Gustavsson, R. , Molin, F. , & Gummesson, S. (2019). Micro‐fossil analysis of Mesolithic human dental calculus, Motala, Sweden‐Indications of health status and paleo‐diet. Journal of Archaeological Science: Reports, 26, 101866.

[ece311053-bib-0076] Oniszczuk, A. , & Podgórski, R. (2015). Influence of different extraction methods on the quantification of selected flavonoids and phenolic acids from *Tilia cordata* inflorescence. Industrial Crops and Products, 76, 509–514.

[ece311053-bib-0077] Ottoni, C. , Borić, D. , Cheronet, O. , Sparacello, V. , Dori, I. , Coppa, A. , Antonović, D. , Vujević, D. , Price, T. D. , Pinhasi, R. , & Cristiani, E. (2021). Tracking the transition to agriculture in Southern Europe through ancient DNA analysis of dental calculus. Proceedings of the National Academy of Sciences of the United States of America, 118(32), e2102116118. 34312252 10.1073/pnas.2102116118 PMC8364157

[ece311053-bib-0078] Oxilia, G. , Bortolini, E. , Badino, F. , Bernardini, F. , Gazzoni, V. , Lugli, F. , Romandini, M. , Radini, A. , Terlato, G. , Marciani, G. , Silvestrini, S. , Menghi Sartorio, J. C. , Thun Hohenstein, U. , Fiorenza, L. , Kullmer, O. , Tuniz, C. , Moggi Cecchi, J. , Talamo, S. , Fontana, F. , … Cristiani, E. (2021). Exploring late Paleolithic and Mesolithic diet in the Eastern Alpine region of Italy through multiple proxies. American Journal of Physical Anthropology, 174, 232–253. 32914870 10.1002/ajpa.24128 PMC7918647

[ece311053-bib-0079] Paknezhad, N. , Mazdarani, F. H. , Hessari, M. , Mobedi, I. , Najafi, F. , Bizhani, N. , Makki, M. , Hassanpour, G. , & Mowlavi, G. (2017). Retrieving ascarid and taeniid eggs from the biological remains of a Neolithic dog from the late 9th millennium BC in Western Iran. Memórias do Instituto Oswaldo Cruz, 112, 593–595. 28902284 10.1590/0074-02760160420 PMC5572444

[ece311053-bib-0080] PalDat . (2019). A palynological database (2000 onwards) . Retrieved April 19, 2022, from https://www.paldat.org/

[ece311053-bib-0081] Payne, R. J. , Lamentowicz, M. , van der Knaap, W. O. , van Leeuwen, J. F. , Mitchell, E. A. , & Mazei, Y. (2012). Testate amoebae in pollen slides. Review of Palaeobotany and Palynology, 173, 68–79.

[ece311053-bib-0082] Perveen, A. (2006). A contribution to the pollen morphology of family Gramineae. World Applied Sciences Journal, 1, 60–65.

[ece311053-bib-0083] Peyron, O. , Goring, S. , Dormoy, I. , Kotthoff, U. , Pross, J. , De Beaulieu, J. L. , Drescher‐Schneider, R. , Vannière, B. , & Magny, M. (2011). Holocene seasonality changes in the central Mediterranean region reconstructed from the pollen sequences of Lake Accesa (Italy) and Tenaghi Philippon (Greece). Holocene, 21, 131–146.

[ece311053-bib-0084] Piro, M. , Mecchia, G. , & Ranieri, C. (2018). Grotta Pila: Elements of geology and new data on anthropic use in protohistoric times. Proceedings of the VII Conference of the Speleological Federation of Lazio, Speleologia del Lazio, 9, 89–94.

[ece311053-bib-0085] Primavera, M. , D'Oronzo, C. , Muntoni, I. M. , Radina, F. , & Fiorentino, G. (2017). Environment, crops and harvesting strategies during the II millennium BC: Resilience and adaptation in socio‐economic systems of Bronze Age communities in Apulia (SE Italy). Quaternary International, 436, 83–95.

[ece311053-bib-0086] Radini, A. , Buckley, S. , Rosas, A. , Estalrrich, A. , De La Rasilla, M. , & Hardy, K. (2016). Neanderthals, trees and dental calculus: New evidence from El Sidrón. Antiquity, 90(350), 290–301.

[ece311053-bib-0087] Radini, A. , Nikita, E. , Buckley, S. , Copeland, L. , & Hardy, K. (2017). Beyond food: The multiple pathways for inclusion of materials into ancient dental calculus. American Journal of Physical Anthropology, 162, 71–83. 28105717 10.1002/ajpa.23147

[ece311053-bib-0108] Radini, A. , Tromp, M. , Beach, A. , Tong, E. , Speller, C. , McCormick, M. , Dudgeon, J. V. , Collins, M. J. , Rühli, F. , Kröger, R. , & Warinner, C. (2019). Medieval women's early involvement in manuscript production suggested by lapis lazuli identification in dental calculus. Science Advances, 5(1), eaau7126. 10.1126/sciadv.aau7126 30662947 PMC6326749

[ece311053-bib-0088] Ramirez, D. A. , Herrera‐Soto, M. J. , Santana‐Sagredo, F. , Uribe‐Rodríguez, M. , & Nores, R. (2021). Parasites in the Atacama Desert: New insights into the lifestyles of ancient human populations (3000–500 BP). Journal of Archaeological Science: Reports, 39, 103171.

[ece311053-bib-0089] Richter, H. G. , Grosser, D. , Heinz, I. , & Gasson, P. E. (2004). IAWA list of microscopic features for softwood identification. IAWA Journal, 25(1), 1–70.

[ece311053-bib-0090] Romboni, M. , Arienzo, I. , Di Vito, M. A. , Lubritto, C. , Piochi, M. , Di Cicco, M. R. , Rickards, O. , Rolfo, M. F. , Sevink, J. , De Angelis, F. , & Alessandri, L. (2023). Isotopic evidence for population dynamics in the Central Italian Copper Age and Bronze Age. PLoS One, 18, e0288637. 37494366 10.1371/journal.pone.0288637 PMC10370757

[ece311053-bib-0091] Rott, E. , Kofler, W. , & Schabetsberger, R. (2009). Ultrastructure of a *Hyalodiscus* species (Bacillariophyceae; Subclass: Coscinodiscophycidae, Fam. Hyalodiscaceae) from brackish waters of Tonga, Oceania. Fottea, 9(2), 299–306.

[ece311053-bib-0092] Rottoli, M. , & Castiglioni, E. (2009). Prehistory of plant growing and collecting in northern Italy, based on seed remains from the early Neolithic to the Chalcolithic (c. 5600–2100 cal BC). Vegetation History and Archaeobotany, 18(1), 91–103.

[ece311053-bib-0093] Scannapicco, F. A. , Bhandary, K. , Ramasubbu, N. , & Levine, M. J. (1990). Structural relationship between the enzymatic and streptococcal binding sites of human salivary α‐amylase. Biochemical and Biophysical Research Communications, 173(3), 1109–1115. 2125215 10.1016/s0006-291x(05)80900-3

[ece311053-bib-0094] Scorrano, G. , Baldoni, M. , Brilli, M. , Rolfo, M. F. , Fornaciari, G. , Rickards, O. , & Martínez‐Labarga, C. (2019). Effect of Neolithic transition on an Italian community: Mora Cavorso (Jenne, Rome). Archaeological and Anthropological Sciences, 11(4), 1443–1459.

[ece311053-bib-0095] Smith, W. W. (1935). The course of stone cell formation in pear fruits. Plant Physiology, 10(4), 587–611. 16653303 10.1104/pp.10.4.587 PMC439172

[ece311053-bib-0096] Smol, J. P. , & Stoermer, E. F. (2010). The diatoms: Applications for the environmental and earth sciences. Cambridge University Press.

[ece311053-bib-0097] Soto, M. , Inwood, J. , Clarke, S. , Crowther, A. , Covelli, D. , Favreau, J. , & Mercader, J. (2019). Structural characterization and decontamination of dental calculus for ancient starch research. Archaeological and Anthropological Sciences, 11, 4847–4872.

[ece311053-bib-0098] Sroka, Z. , & Bełz, J. (2009). Antioxidant activity of hydrolyzed and non‐hydrolyzed extracts of the inflorescence of linden (*Tiliae inflorescentia*). Advances in Clinical and Experimental Medicine, 18(4), 329–335.

[ece311053-bib-0099] Stevens, C. J. , Murphy, C. , Roberts, R. , Lucas, L. , Silva, F. , & Fuller, D. Q. (2016). Between China and South Asia: A Middle Asian corridor of crop dispersal and agricultural innovation in the Bronze Age. Holocene, 26(10), 1541–1555. 27942165 10.1177/0959683616650268 PMC5125436

[ece311053-bib-0100] Stone, J. R. , & Yost, C. L. (2020). Diatom microfossils in archaeological settings. In A. Henry (Ed.), Handbook for the analysis of micro‐particles in archaeological samples (pp. 23–64). Springer.

[ece311053-bib-0101] Suzuki, N. , & Not, F. (2015). Biology and ecology of radiolaria. In S. Ohtsuka , T. Suzaki , T. Horiguchi , N. Suzuki , & F. Not (Eds.), Marine protists (pp. 179–222). Springer.

[ece311053-bib-0102] Tafuri, M. A. , Craig, O. E. , & Canci, A. (2009). Stable isotope evidence for the consumption of millet and other plants in Bronze Age Italy. American Journal of Physical Anthropology, 139(2), 146–153. 19051259 10.1002/ajpa.20955

[ece311053-bib-0103] Ucchesu, M. , Manunza, M. R. , & Sabato, D. (2018). Agriculture and exploitation of wild plants at Chalcolithic (4th to 3rd millennium cal BC) sites in Sardinia (Italy). Archaeological and Anthropological Sciences, 10(7), 1693–1702.

[ece311053-bib-0104] Varalli, A. , Moggi‐Cecchi, J. , & Goude, G. (2022). A multi‐proxy bioarchaeological approach reveals new trends in Bronze Age diet in Italy. Scientific Reports, 12(1), 1–20. 35842420 10.1038/s41598-022-15581-0 PMC9288517

[ece311053-bib-0105] Wang, T. , Wei, D. , Jiang, Z. , Xia, X. , Wu, Y. , Han, Z. , & Fuller, B. T. (2022). Microfossil analysis of dental calculus and isotopic measurements reveal the complexity of human‐plant dietary relationships in Late Bronze Age Yunnan. Archaeological and Anthropological Sciences, 14, 94.

[ece311053-bib-0106] Warinner, C. , Hendy, J. , Speller, C. , Cappellini, E. , Fischer, R. , Trachsel, C. , & Collins, M. J. (2014). Direct evidence of milk consumption from ancient human dental calculus. Scientific Reports, 4, 7104. 25429530 10.1038/srep07104 PMC4245811

[ece311053-bib-0107] Zeder, M. A. (2008). Domestication and early agriculture in the mediterranean basin: Origins, diffusion, and impact. Proceedings of the National Academy of Sciences of the United States of America, 105, 11597–11604. 10.1073/pnas.0801317105 18697943 PMC2575338

