# Supplementary material for: Microparticles from dental calculus disclose paleoenvironmental and palaeoecological records
Source: Ecol Evol. 2024 Feb 23;14(2):e11053. doi: 10.1002/ece3.11053 (PMC10891416; doi:10.1002/ece3.11053)
Supplement: Supplementary file 1 — Appendix S1 [file ECE3-14-e11053-s002.docx]

**SUPPLEMENTAL MATERIAL 0.**

**SM0. Radiocarbon dates from La Sassa and Pila archaeological sites.** Dated material (sample type), stratigraphic details (SU, stratigraphic unit) about the contexts, and radiocarbon dates calibrated with the software OxCal (version 4.4.4- Bronk Ramsey, 2021) using the IntCal20 curve (Reimer et al., 2020).

| **Archaeological site** | **Sample** **type** | **Context** | **Radiocarbon dates (^14^C)** | **Calibrated age** |
| --- | --- | --- | --- | --- |
| **La Sassa** | Human femur | Room 1, SU 97 | 3316±45 | 3116-2919 calBC |
|  | Human femur | Room 2, SU 19 | 4000±35 | 2585-2458 calBC |
|  | Human femur | Room 1, SU 55 | 3722±40 | 2210-2017 calBC |
|  | Human femur | Room 1, SU 78 | 3451±45 | 1885-1630 calBC |
|  | *Sus* sp., right ulna | Room 1, SU 9 | 3148±45 | 1517-1375 calBC |
| **Pila** | Human bone | Saggio B | 4175± 35 BP | 2886 – 2662 calBC |
|  | Human bone | Saggio B | 3775± 50 BP | 2349 – 2031 calBC |

**REFERENCES**

Bronk Ramsey C. 2021. OxCal 4.4.4. Retrieved from: <http://c14.arch.ox.ac.uk/oxcal> (May 2023).

Reimer, P. J., Austin, W. E., Bard, E., Bayliss, A., Blackwell, P. G., Ramsey, C. B., Butzin, M., Cheng, H., Edwards, R., Friedrich, M., Grootes, P., Guilderson, T., Hajdas, I., Heaton, T., Hogg, A., Hughen, K., Kromer, B., Manning, S., Muscheler, R., … Talamo, S. (2020). The IntCal20 Northern Hemisphere radiocarbon age calibration curve (0–55 cal kBP). Radiocarbon, 62, 725–757.
