# Supplementary material for: Microparticles from dental calculus disclose paleoenvironmental and palaeoecological records
Source: Ecol Evol. 2024 Feb 23;14(2):e11053. doi: 10.1002/ece3.11053 (PMC10891416; doi:10.1002/ece3.11053)
Supplement: Supplementary file 2 — Appendix S2 [file ECE3-14-e11053-s001.docx]

**SUPPLEMENTARY INFORMATION 1 (SI1). Microparticles from calculi.**

**SI1-Table 1. Plant microparticles recovered from dental calculi of human samples buried in La Sassa cave (LSC) archaeological site.** Amount and proposed identification of microdebris, detected by optic microscopy, were reported for each specimen (isolated teeth: 1-63; single individuals: A-Q). Teeth numbering is according to the International Dental Federation tooth notation ISO 3950. Legend for calculus location: M, molar; P, premolar; ND, not determined. Legend: Morphotype I, Triticeae starch granules; Morphotype II, Panicoideae starch granules; As, Asteroideae pollen; B/O, Brassicaceae/Oleaceae pollen; Be, Betulaceae pollen (Sub Family Coryloideae, *Ostrya carpinifolia* or Sub Family Betuloideae, *Betula* sp.); BeA, Betulaceae, Betuloideae, *Alnus* pollen; F, Fagaceae, *Quercus* deciduous pollen; GC, Gimnosperm Cupressaceae pollen; MT, Malvaceae, Subfamily Tilioideae (*Tilia* pollen); P: Poaceae spontaneous group pollen; ND, pollen not determined; Br, brachysclereid aggregates; SP, sporangium; Tr, trichome.

| **Lab Code** | **Calculus location (teeth)** | **Starch morphotype** | | **Not determined starch** | **Total starches per sample** | **Pollen and spores** | **NPPs** |
| --- | --- | --- | --- | --- | --- | --- | --- |
|  |  | **I** | **II** |  |  |  |  |
| **LSC 1** | 22 | 7 |  |  | **7** |  |  |
| **LSC 2** | 13 |  |  |  | **0** |  |  |
| **LSC 3** | 21 |  |  |  | **0** |  |  |
| **LSC 4** | 31 |  |  |  | **0** |  |  |
| **LSC 5** | 12,14-17,24-27 |  |  |  | **0** |  |  |
| **LSC 6** | 45,46 |  |  |  | **0** |  |  |
| **LSC 7** | 16,17 |  |  | 2 | **2** | 1 BeA |  |
| **LSC 8** | 27 |  |  |  | **0** |  |  |
| **LSC 9** | 23 |  |  |  | **0** |  |  |
| **LSC 10** | 27 |  |  |  | **0** |  |  |
| **LSC 11** | 27 |  |  | 1 | **1** |  |  |
| **LSC 12** | 13 |  |  |  | **0** |  |  |
| **LSC 13** | 11,22 |  |  |  | **0** |  |  |
| **LSC 14** | 31,32 |  |  |  | **0** |  |  |
| **LSC 15** | 23,23 |  |  |  | **0** |  |  |
| **LSC 16** | 33 |  |  |  | **0** |  |  |
| **LSC 17** | 14 |  |  |  | **0** |  |  |
| **LSC 18** | 34 |  |  |  | **0** |  |  |
| **LSC 19** | 45 |  | 2 |  | **2** |  |  |
| **LSC 20** | 45 |  |  |  | **0** |  |  |
| **LSC 21** | 35 |  |  |  | **0** |  |  |
| **LSC 22** | 27 |  |  |  | **0** |  |  |
| **LSC 23** | 38 |  |  |  | **0** |  |  |
| **LSC 24** | 48 |  |  |  | **0** |  |  |
| **LSC 25** | 16 |  |  |  | **0** |  |  |
| **LSC 26** | 24 |  |  |  | **0** |  |  |
| **LSC 27** | M nd | 1 |  |  | **1** |  |  |
| **LSC 28** | 16 |  |  |  | **0** | 1 P |  |
| **LSC 29** | 11,12 |  | 2 |  | **2** |  |  |
| **LSC 30** | 41 |  |  |  | **0** |  |  |
| **LSC 31** | 38 |  |  |  | **0** |  |  |
| **LSC 32** | 18 |  |  |  | **0** |  |  |
| **LSC 33** | 26 |  |  |  | **0** |  |  |
| **LSC 34** | 17 |  |  |  | **0** |  |  |
| **LSC 35** | 11,21 |  |  |  | **0** |  |  |
| **LSC 36** | 45 |  |  |  | **0** |  |  |
| **LSC 37** | 43 |  |  |  | **0** |  |  |
| **LSC 38** | P nd |  | 1 |  | **1** |  |  |
| **LSC 39** | 24,25 |  |  | 2 | **2** |  |  |
| **LSC 40** | 45 |  |  |  | **0** |  |  |
| **LSC 41** | 11,21 |  |  | 1 | **1** |  |  |
| **LSC 42** | 27 |  |  |  | **0** | 1 MT |  |
| **LSC 43** | 13 |  |  |  | **0** | 1 ND |  |
| **LSC 44** | 38 |  |  |  | **0** |  |  |
| **LSC 45** | 24 |  |  |  | **0** |  |  |
| **LSC 46** | 24 |  |  |  | **0** |  |  |
| **LSC 47** | 41 |  |  |  | **0** |  |  |
| **LSC 48** | 16 |  |  |  | **0** |  |  |
| **LSC 49** | 22 |  | 2 | 1 | **3** |  |  |
| **LSC 50** | 11,21 |  |  |  | **0** |  |  |
| **LSC 51** | 17 |  |  |  | **0** |  |  |
| **LSC 52** | 23 |  | 4 |  | **4** |  |  |
| **LSC 53** | 31 |  |  | 1 | **1** |  |  |
| **LSC 54** | 13 |  | 1 |  | **1** |  |  |
| **LSC 55** | 15 |  |  |  | **0** |  |  |
| **LSC 56** | P nd | 1 | 23 |  | **24** |  |  |
| **LSC 57** | 31 |  |  |  | **0** |  |  |
| **LSC 58** | 41 |  |  |  | **0** |  |  |
| **LSC 59** | 24 |  | 2 |  | **2** |  |  |
| **LSC 60** | 43 |  |  |  | **0** |  |  |
| **LSC 61** | 25,26 |  |  |  | **0** | 1 F |  |
| **LSC 62** | 22 |  |  |  | **0** |  |  |
| **LSC 63** | 33 |  | 12 | 4 | **16** |  | 1 Tr |
| **LSC A** | 33,35,37,38 |  | 14 |  | **14** | 1 ND |  |
| **LSC B** | 32,33,34,48 | 1 | >51 |  | **>52** |  |  |
| **LSC C** | 41,43,44 |  | 18 | 5 | **23** | 1 GC |  |
| **LSC D** | 37 |  | 19 | 3 | **22** | 1 Be |  |
| **LSC E** | 43-45,47,48 |  | 31 |  | **31** |  |  |
| **LSC F** | 34,36,37,42,43,46,48 | >843 | 11 |  | **>854** |  |  |
| **LSC G** | 34,38,47,48 |  | 13 |  | **13** |  |  |
| **LSC H** | 22,31,33,42 |  | 7 |  | **7** |  |  |
| **LSC I** | 34,37,44-48 |  | 85 | 5 | **90** | 2 GC |  |
| **LSC L** | 14,15,46 |  | 12 |  | **12** | 1B/O, 2 F |  |
| **LSC M** | 13,26,41,45,47 |  | 40 | 1 | **41** |  |  |
| **LSC N** | 43-45 | 3 | 46 | 4 | **53** |  | 19 Br |
| **LSC O** | 12-17,23,26, 41-45 | 1 | 33 | 3 | **37** | 3As, 1 SP | 2 Br |
| **LSC P** | 17 |  | 10 |  | **10** |  |  |
| **LSC Q** | 23,47 |  | 37 |  | **37** |  |  |
| **TOTAL** |  | **>857** | **>476** | **33** | **>1366** | **16** | **22** |

**SI1-Table 2. Microparticles recovered from dental calculi of human samples found in Pila cave (PC) archaeological site.** Amount and proposed identification of microfossils detected by optic microscopy were reported for each specimen (isolated teeth: 1-30; single individuals: A-I). Teeth numbering is according to the International Dental Federation tooth notation ISO 3950. Legend for calculus location: M, molar; P, premolar; ND, not determined. Legend for starch and pollen: Morphotype I, Triticeae starch granules; Morphotype II, Panicoideae starch granules; As, Asteraceae pollen; B, Betulaceae pollen (Sub Family Coryloideae, *Ostrya carpinifolia* or Sub Family Betuloideae, *Betula* sp.); Fb, Fabaceae pollen; F, Fagaceae (*Castanea* sp. or *Quercus* deciduous pollen); GC, Gimnosperm Cupressaceae pollen; MT, Malvaceae, Subfamily Tilioideae (*Tilia* pollen); O, Oleaceae pollen; P: Poaceae pollen; Tf, *Trifolium* pollen; U, Ulmaceae, *Ulmus* pollen; ND, pollen not determined. Legend for non-pollen palynomorphs (NPPs): Cm, charred material (not completely carbonized) containing articulate phytoliths; L, Lepidoptera wing fragment; Pe, parasite eggs; Ph, phytolith; Pt, plant tissue; Ta: putative amoebae; Tpm, tracheid pitting more seriate; Tpu, tracheid pitting uniseriate.

| **Lab Code** | **Calculus location (teeth)** | **Starch Morphotype** | | **Not determined starch** | **Total starches per sample** | **Pollen** | **Fern spores** | **NPPs** | | |
| --- | --- | --- | --- | --- | --- | --- | --- | --- | --- | --- |
|  |  | **I** | **II** |  |  |  |  | **Palynodebris elements** | **Animal and protist microdebris** | **Fragments of diatoms and/or radiolarians** |
| **PC 1** | 21 | 3 | 366 | 6 | **375** | 6 F, 1 ND |  |  |  |  |
| **PC 2** | M nd | 85 | 919 |  | **1004** | 8 F |  |  |  |  |
| **PC 3** | 33 | 9 | 1423 | 7 | **1439** | 22 F, 1 GC, 3 ND |  |  |  |  |
| **PC 4** | 24 | 1 | 279 | 5 | **285** | 3 F |  |  |  |  |
| **PC 5** | 14 |  | 259 | 12 | **271** | 7 F, 1 MT, 2 ND | 1 |  |  |  |
| **PC 6** | 46 | 1 | 477 | 5 | **483** | 9 F, 3 ND |  |  |  |  |
| **PC 7** | P nd | 86 | 210 | 37 | **333** | 6 F, 1 ND |  |  |  |  |
| **PC 8** | P nd | 20 | 313 | 9 | **342** | 8 F, 1 U |  |  |  | 1 |
| **PC 9** | 15 | 1 | 654 | 214 | **869** | 19 F, 4 ND |  |  |  |  |
| **PC 10** | 34 | 39 | 72 |  | **111** | 1 F, 1 GC, 1 ND |  |  |  |  |
| **PC 11** | 25 |  | 503 | 37 | **540** | 17 F, 1 ND |  |  |  | 2 |
| **PC 12** | 23 |  | 281 | 17 | **298** | 11 F, 1 ND |  | 1 Pt | 58 Pe | 3 |
| **PC 13** | M nd | 3 | 106 | 235 | **344** | 3 F, 6 ND |  | 3 Pt, 1 Tpu |  | 5 |
| **PC 14** | 31 | 5 | 294 | 2 | **301** | 12 F, 1 GC, 7 ND |  |  |  | 11 |
| **PC 15** | 21 | 16 | 974 | 33 | **1023** | 24 F, 7 ND | 1 | 2 Pt, 1 Tpm |  | 1 |
| **PC 16** | 23 | 10 | 335 | 17 | **362** | 36 F, 2 ND |  |  |  | 2 |
| **PC 17** | P nd | 170 | 106 | 5 | **281** | 5 ND |  |  |  | 1 |
| **PC 18** | M nd | 12 | 390 | 7 | **409** | 1 As, 7 F, 11 ND |  | 2 Pt |  | 4 |
| **PC 19** | 27 | 6 | 156 | 117 | **279** | 10 F, 6 ND |  |  |  | 1 |
| **PC 20** | P nd | 4 | 257 | 1 | **262** | 19 F, 1 P, 2 ND |  | 1 Pt, 2 Tpu |  | 3 |
| **PC 21** | P nd | 4 | 176 | 22 | **202** | 4 F |  |  |  |  |
| **PC 22** | 45 | 20 | 403 | 32 | **455** | 31 F, 2 GC |  |  |  |  |
| **PC 23** | 25 | 66 | 267 | 4 | **337** | 1 F, 6 ND |  |  |  | 1 |
| **PC 24** | M nd | 29 | 1015 | 42 | **1086** | 6 F, 1 GC, 1 ND |  | 1 Pt |  | 2 |
| **PC 25** | 31 | 30 | 470 | 48 | **548** | 5 F, 1 ND |  | 1 Ph, 2 Pt, 1 Tpm | 1 L, 1 TA | 5 |
| **PC 26** | 26 | 6 | 312 | 5 | **323** | 24 F, 2 ND |  |  |  |  |
| **PC 27** | 24 | 15 | 441 | 5 | **461** | 8 F, 3 GC |  | 1 Ph, 1 Pt |  |  |
| **PC 28** | M nd | 30 | 263 | 10 | **303** | 4 F, 1 O |  |  |  |  |
| **PC 29** | M nd | 1 | 165 | 21 | **187** | 3 F, 1 Fb |  |  |  |  |
| **PC 30** | M nd | 12 | 360 | 5 | **377** | 1 B, 8 F, 1 GC, 4 Tf, 1 ND |  | 1 Pt |  |  |
| **PC A** | 17  16 | 39 | 420 | 49 | **508** | 21 F, 4 ND |  | 2 Pt |  |  |
| **PC B** | 22  31 | 21 | 606 | 25 | **652** | 25 F, 1 ND |  |  |  |  |
| **PC C** | 16  17 | 38 | 1074 | 25 | **1137** | 29 F, 1 GC, 2 ND |  |  |  | 5 |
| **PC D** | 11  17,16  M nd  M nd | 16 | 182 | 6 | **204** | 3 F, 1 GC |  | 1 Pt |  | 2 |
| **PC E** | 43,44 | 13 | 339 | 10 | **362** | 6 F |  |  |  | 3 |
| **PC F** | 43,46  31,32,41,42 | 54 | 752 | 20 | **826** | 18 F, 1 GC |  | 3 Cm, 5 Pt |  | 4 |
| **PC G** | 44,45 | 18 | 596 | 17 | **631** | 22 F, 1 ND |  |  |  |  |
| **PC H** | 46 | 8 | 369 | 56 | **433** | 1 As, 12 F |  | 1 Pt | 1 TA |  |
| **PC I** | 46,47,48 | 7 | 396 | 19 | **422** | 31 F, 2 GC |  | 1 Cm, 1 Ph |  | 2 |
| **TOTAL** |  | **898** | **16980** | **1187** | **19065** | **598** | **2** | **35** | **61** | **58** |
